# Integrin signaling is critical for myeloid-mediated support of T-cell acute lymphoblastic leukemia

**DOI:** 10.1038/s41467-023-41925-z

**Published:** 2023-10-07

**Authors:** Aram Lyu, Ryan S. Humphrey, Seo Hee Nam, Tyler A. Durham, Zicheng Hu, Dhivya Arasappan, Terzah M. Horton, Lauren I. R. Ehrlich

**Affiliations:** 1 Department of Molecular Biosciences, The University of Texas at Austin, Austin, TX USA; 2 Bakar Computational Health Sciences Institute, University of California, San Francisco, San Francisco, CA USA; 3 Center for Biomedical Research Support, The University of Texas at Austin, Austin, TX USA; 4 Department of Pediatrics, Baylor College of Medicine/Dan L. Duncan Cancer Center and Texas Children’s Cancer Center, Houston, TX USA; 5 Department of Oncology, Livestrong Cancer Institutes, The University of Texas at Austin Dell Medical School, Austin, TX USA

**Keywords:** Acute lymphocytic leukaemia, Cancer microenvironment

## Abstract

We previously found that T-cell acute lymphoblastic leukemia (T-ALL) requires support from tumor-associated myeloid cells, which activate Insulin Like Growth Factor 1 Receptor (IGF1R) signaling in leukemic blasts. However, IGF1 is not sufficient to sustain T-ALL in vitro, implicating additional myeloid-mediated signals in leukemia progression. Here, we find that T-ALL cells require close contact with myeloid cells to survive. Transcriptional profiling and in vitro assays demonstrate that integrin-mediated cell adhesion activates downstream focal adhesion kinase (FAK)/ proline-rich tyrosine kinase 2 (PYK2), which are required for myeloid-mediated T-ALL support, partly through activation of IGF1R. Blocking integrin ligands or inhibiting FAK/PYK2 signaling diminishes leukemia burden in multiple organs and confers a survival advantage in a mouse model of T-ALL. Inhibiting integrin-mediated adhesion or FAK/PYK2 also reduces survival of primary patient T-ALL cells co-cultured with myeloid cells. Furthermore, elevated integrin pathway gene signatures correlate with higher FAK signaling and myeloid gene signatures and are associated with an inferior prognosis in pediatric T-ALL patients. Together, these findings demonstrate that integrin activation and downstream FAK/PYK2 signaling are important mechanisms underlying myeloid-mediated support of T-ALL progression.

## Abstract (web summary)

Tumor-associated myeloid cells directly support progression of T-cell acute lymphoblastic leukemia (TALL). Here, the authors show that T-ALL cells must contact myeloid cells and activate integrin signaling and downstream FAK/PYK2 kinases to survive.

## Introduction

T-cell acute lymphoblastic leukemia (T-ALL) is a hematologic malignancy arising from neoplastic transformation of T-cell progenitors. ALL is the most common pediatric malignancy, with T-ALL accounting for ~15% and ~25% of pediatric and adult ALL cases, respectively^1^. T-ALL is associated with infiltration of leukemic blasts into multiple organs, including the spleen, bone marrow (BM), lymph nodes (LN), liver, and central nervous system^2^. Although current intensified chemotherapy regimens have significantly improved prognoses, with 5-year survival rates for pediatric T-ALL exceeding 90%^3,4^, these therapies are toxic and are associated with long-term morbidities, such as neurological toxicity, cognitive impairment, and metabolic disorders^5,6^. Moreover, the survival rate for relapsed patients is < 25%^7^. Therefore, identifying targets for development of less toxic and more effective therapies would be clinically beneficial. Multiple genetic drivers of T-ALL, including activating mutations in *NOTCH1*, deletion of the *CDKN2A* tumor suppressor locus, and aberrant expression of transcription factors, such as T-cell acute lymphocytic leukemia 1 (TAL1) and LIM domain only 2 (LMO2), have been identified^8–11^. Additionally, unbiased genome-wide profiling analyses have expanded our understanding of the genetic landscape of T-ALL, identifying novel driver genes and associated molecular mechanisms that promote leukemia initiation and progression^12^. However, despite multiple pro-leukemic genomic lesions, T-ALL cells cannot survive in vitro in the absence of cytokines or supportive cell types^13,14^, suggesting that cells or signals in the leukemia-microenvironment are required to support leukemia growth and could thus serve as therapeutic targets.

The tumor microenvironment (TME), which is shaped by interactions between tumor cells and their non-transformed neighbors, plays an essential role in promoting the progression of both solid tumors and hematologic malignancies^15,16^. Several cell types in the TME have been shown to support T-ALL survival and progression, including vascular endothelial cells through a C-X-C motif chemokine receptor 4 (CXCR4)-CXCL12 axis in the BM^17,18^, and thymic epithelial cells (TECs) through release of interleukin 7 (IL-7) and expression of NOTCH1 ligands^9,19,20^. We have previously shown that tumor-associated myeloid cells provide critical support for T-ALL, in part by secreting IGF1 to activate IGF1R signaling in leukemic cells^13,14^. However, IGF1 is not sufficient to support T-ALL survival in culture, and myeloid cells sensitize T-ALL cells to IGF1^14^, suggesting a role for additional molecular mechanisms underlying myeloid-mediated T-ALL support. We have previously shown that relative to healthy thymocytes, T-ALL cells make frequent and prolonged contact with DCs in the thymus^13^, suggesting physical interactions could play a critical role in myeloid-mediated T-ALL support.

Integrins are known mediators of cellular interactions; there are 24 heterodimeric integrin receptors in humans, each consisting of an α and β chain^21^. Integrins bind a variety of ligands, including extracellular matrix (ECM) components like fibronectin, collagen, and laminin, as well as adhesion molecules like intercellular adhesion molecule 1 (ICAM-1) and vascular cell adhesion molecule 1 (VCAM-1), expressed by antigen-presenting cells (APCs) and endothelial cells^22^. Upon ligand binding, integrins undergo a conformational change, adopting a high-affinity form that recruits cytoskeletal structures via talin and other linker proteins^23^. Ligand-bound integrins activate downstream kinases, including FAK and PYK2, leading to diverse cellular outcomes including cell migration, survival, and proliferation^23^. Integrin-mediated interactions with stromal cells or APCs are essential for T-cell development in the thymus and T-cell homeostasis, migration, and activation in the periphery^24–26^. Notably, integrins signal cooperatively with growth factor receptors, including IGF1R^23,27,28^, and dysregulated integrin signaling has been shown to promote tumorigenesis, metastasis, and chemotherapeutic resistance in a variety of cancers^23,29^. Tumor cells can disrupt local ECM and recruit host cells that express integrin ligands, inducing hyperactivated integrin signaling^30,31^. Given that integrin signaling promotes survival of healthy T cells and tumors, along with our previous observation that T-ALL cells make frequent and prolonged contact with myeloid cells in the thymic TME^13^, we hypothesized that integrin signaling might promote myeloid-mediated support of T-ALL.

Here, we report that survival of primary mouse T-ALL cells is dependent on close contact with tumor-associated myeloid cells. Transcriptional profiling revealed elevated expression of integrins and enrichment of cell-cell adhesion pathways in T-ALL cells, suggesting integrins could promote adhesion to myeloid cells. Consistent with this possibility, inhibiting integrin-mediated cell adhesion between T-ALL and myeloid cells diminishes IGF1R activation and results in a significant decrease in T-ALL survival in vitro. Notably, blockade of ICAM-1 and/or VCAM-1 in leukemic mice, or downstream FAK/PYK2 pathways, significantly diminishes T-ALL burden in multiple organs and confers a significant survival benefit in leukemic mice. Consistent with results from mouse models, integrin-mediated cell adhesion and FAK/PYK2 signaling are required for myeloid-mediated support of primary T-ALL cells from pediatric patients. Elevated integrin pathway gene signatures are also associated with an enrichment of myeloid gene signatures and inferior outcomes in pediatric T-ALL patients. Collectively, these results reveal that tumor-associated myeloid cells promote leukemia survival and progression by activating integrin signaling in mouse models of T-ALL and in primary human T-ALL.

## Results

### Close contact is required for myeloid-mediated T-ALL support and integrins are candidate mediators of these interactions

**Fig. 1:**
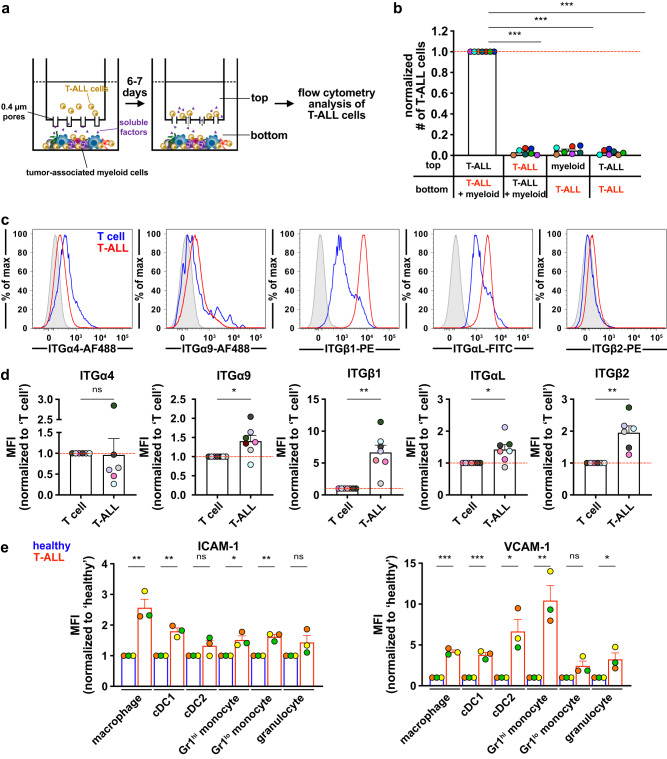
Close contact is critical for myeloid-mediated T-ALL survival, and integrin expression is elevated on T-ALL cells. **a** Schematic illustration of transwell assays in which LN3 T-ALL cells are cultured in the presence or absence of enriched tumor-associated myeloid cells in the same or opposite chambers to determine whether T-ALL cells require close contact with myeloid cells to survive. **b** Quantification of viable T-ALL cells in the chamber indicated in red 6–7 days after co-culture initiation. Results were normalized to the viability of T-ALL cells making physical contact with tumor-associated myeloid cells in the bottom chamber within each experiment. Bars depict the mean + SEM of cumulative data from *n* = 7 experiments, each with a distinct color-coded primary T-ALL; symbols represent the mean of 2-3 replicate wells per experiment. **c** Representative flow cytometry plots and (**d**) quantification ofcell surface integrin expression levels of transplanted LN3 T-ALL cells (red; CD45.2^+^CD5^+^) and host T cells (blue; CD45.1^+^CD5^+^) from the same leukemic spleens, quantified as median fluorescence intensity (MFI) values by flow cytometry. Isotype control stains are shaded in gray. **d** Results were normalized to T-cell levels within each experiment. Data are compiled from *n* = 6 (ITGα4, ITGβ2) or 7 (ITGα9, ITGβ1, ITGαL) independent experiments, each with a distinct color-coded primary T-ALL; symbols represent individual mice. **e** Quantification of ICAM-1 (left) and VCAM-1 (right) protein levels on the indicated myeloid subsets from the spleens of healthy (blue) or leukemic (red) mice. Results were normalized to healthy controls within each subset. Bars depict the mean + SEM of cumulative data from *n* = 3 experiments, each with a distinct color-coded primary T-ALL; symbols represent individual mice. Statistical significance was determined by (**b**) a two-sided repeated measures one-way ANOVA with the Holm-Sidak correction, (**d**) two-sided paired Student *t*-tests, and (**e**) two-sided unpaired Student *t*-tests; *P*-values: *<0.05, **<0.01, ***<0.001. ns, not significant. Source data are provided as a Source Data file.

We previously found that tumor-associated myeloid cells support survival of T-ALL cells, but not healthy T cells^14^. To identify candidate mechanisms underlying the preferential ability of T-ALL cells to receive myeloid support, we re-evaluated our published RNA-seq datasets of primary LN3 mouse T-ALL cells and healthy T-lineage cells from the thymus and spleen (GSE150096)^14^. Gene ontology analysis showed that gene signatures of “cell-cell adhesion” were significantly enriched in thymic T-ALL cells relative to healthy thymocytes (*q*-value = 0.015; Supplementary Fig. 1a). To test whether T-ALL cells must physically interact with tumor-associated myeloid cells to survive, we engrafted non-conditioned CD45 congenic mice with primary LN3 T-ALL cells^32^. Once the mice became leukemic, splenic T-ALL cells were cultured in the presence or absence of enriched tumor-associated myeloid cells (Supplementary Fig. 1b) in transwell plates, such that the two cell types could either contact one another or were separated by micropores through which only soluble factors could pass (Fig. 1a). Notably, T-ALL cells survived only when co-cultured in the same chamber with tumor-associated myeloid cells (Fig. 1b), indicating cellular contact is required for myeloid cells to support T-ALL survival. To identify potential mediators of physical interactions, we carried out further transcriptional analyses between thymic T-ALL cells versus healthy thymocytes and found that the “Integrin1” pathway was preferentially enriched in T-ALL cells (Supplementary Fig. 2a, b), and ITGβ1 transcript and protein levels were elevated in T-ALL cells (Fig. 1c, d, Supplementary Fig. 2c). Among ITGβ1 heterodimers, ITGα4β1 (VLA-4) and ITGα9β1 mediate cell-cell interactions via binding to VCAM-1^22^. Both the transcript and protein levels of ITGα9, but not ITGα4, were significantly higher in T-ALL cells relative to controls (Fig. 1c, d, Supplementary Fig. 2d). The “ILK (Integrin-linked kinase)” pathway was also enriched in T-ALL relative to healthy CD8^+^ T cells in the spleen (Supplementary Fig. 2e, f), with elevated levels of ILK in T-ALL cells (Supplementary Fig. 2g, h). Activation of ILK is associated with ITGαLβ2 (LFA-1) signaling, and LFA-1 promotes T-cell activation upon binding its ligand ICAM-1 on APCs^26^. Thus, we investigated expression of LFA-1 by T-ALL cells and found it was significantly elevated in T-ALL blasts relative to T cells in the spleen (Fig. 1c, d), despite diminished transcript levels (Supplementary Fig. 2i). To test the possibility that our findings from the comparison between T-ALL cells and healthy CD8^+^ T cells in the spleen (Fig. 1c, d and Supplementary Fig. 2e–h) could reflect developmental differences between immature thymocytes, from which T-ALL arises, and splenic T cells, we compared the transcriptomes of healthy thymocytes to healthy splenic CD8^+^ T cells, and found that integrin-associated pathways were not enriched in thymocytes (Supplementary Fig. 3a). We also evaluated expression levels of integrins on healthy splenic T cells, healthy thymocytes, and splenic T-ALL cells. T-ALL cells expressed elevated levels of ITGα9β1 and LFA-1 relative to both thymocytes and splenic CD8^+^ T cells (Supplementary Fig. 3b, c). We next tested whether the integrin ligands ICAM-1 and VCAM-1 were expressed by tumor-associated myeloid subsets, with a particular interest in macrophages, monocytes, and cDC2s, the myeloid cell types we previously found promote T-ALL survival^14^. While tumor-associated macrophages, cDC2s and monocytes all express ICAM-1, only tumor-associated macrophages express high levels of VCAM-1 (Supplementary Fig. 4a, b). Notably, both ICAM-1 and VCAM-1 protein levels were elevated in tumor-associated myeloid subsets relative to those in healthy littermate controls (Fig. 1e, Supplementary Fig. 4b), which could contribute to the superior leukemia-supportive capabilities of tumor-associated myeloid cells^13,14^. Further transcriptional analysis revealed that expression of proinflammatory cytokines, including *Tnf* and *Il1b*, which are known NF-κB activators that upregulate adhesion molecules^33^, was comparable between T-ALL cells and healthy T cells in the thymus and spleen (Supplementary Fig. 4c), suggesting T-ALL-mediated activation of NF-κB signaling in tumor-associated myeloid cells is not a likely mechanism for upregulation of adhesion molecules. Prior studies reported that leukemia-supportive niches, including the BM^34^, are hypoxic, causing oxidative stress that upregulates adhesion molecules^35^. *Hif1a* transcript levels were significantly higher in T-ALL cells in both organs (Supplementary Fig. 4c), implicating hypoxia as a potential mechanism underlying elevated ICAM-1 and VCAM-1 levels in tumor-associated myeloid cells. Collectively, these results demonstrate that close contact between T-ALL cells and tumor-associated myeloid cells is critical for leukemia survival and identify integrins as candidate mediators of these interactions.

### Inhibiting integrin-mediated cell adhesion diminishes myeloid support of T-ALL by reducing IGF1R signaling

**Fig. 2:**
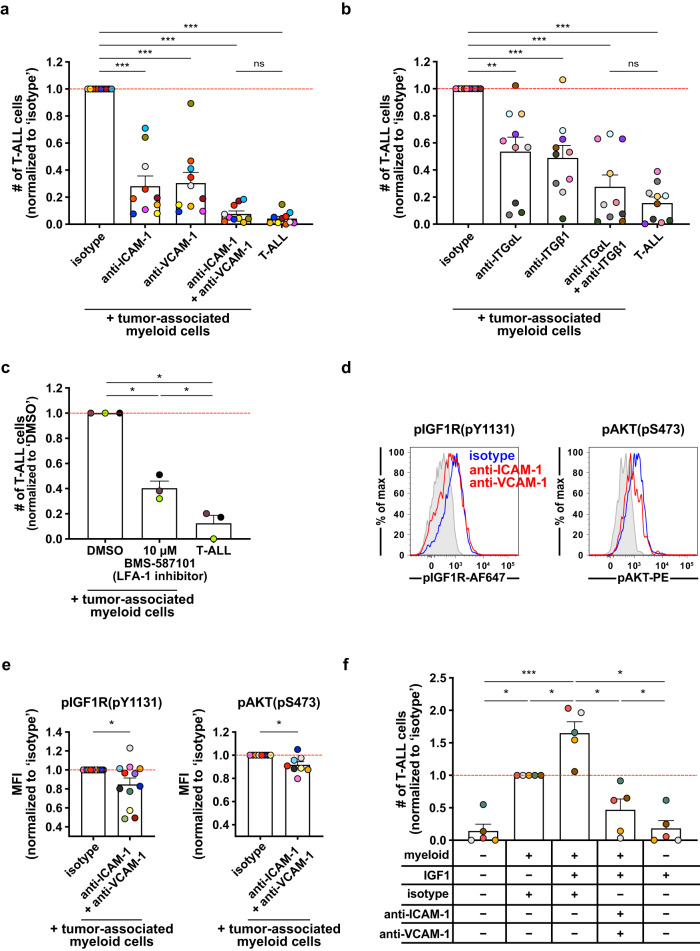
Integrin-mediated cell adhesion is required for myeloid support of T-ALL survival in vitro and for sensitization of T-ALL cells to IGF1R signaling. **a**, **b** Quantification of viable splenic LN3 T-ALL cells 6–7 days after co-culture with tumor-associated myeloid cells in the presence of (**a**) anti-ICAM-1 and/or anti-VCAM-1 blocking antibodies or (**b**) anti-integrin αL (ITGαL) and/or anti-ITGβ1 blocking antibodies (10 µg/ml for each). Results were normalized to isotype-treated cultures. Bars show mean + SEM from *n* = 10 independent experiments, with distinct color-coded primary T-ALL; symbols represent averages of 2-3 technical replicate wells. Red lines indicate normalized mean viability of isotype-treated T-ALL cells. **c** Quantification of viable splenic LN3 T-ALL cells 6–7 days after co-culture with tumor-associated myeloid cells +/- 10 µM BMS-587101 (LFA-1 inhibitor) or DMSO control. Results were normalized to DMSO-treated cultures. Bars show mean + SEM from *n* = 3 independent experiments, with distinct color-coded primary T-ALL; symbols represent averages of 2–3 technical replicate wells. The red line indicates normalized mean viability of DMSO-treated T-ALL. **d**, **e** (**d**) Representative flow cytometry plots and  (**e**) quantification of phosphorylated (p)IGF1R and pAKT in LN3 T-ALL cells co-cultured with tumor-associated myeloid cells for 4–5 days with anti-ICAM-1 and anti-VCAM-1 blocking antibodies (red in (**d**); 10 µg/ml each) or isotype controls (blue in (**d**)). Isotype control stains are shaded in gray in (**d**). Results in (**e**) were normalized to the MFI of isotype-treated cultures in each experiment. Bars represent mean + SEM from *n* = 8 (pAKT) or *n* = 12 (pIGF1R) independent experiments, each with a distinct color-coded primary T-ALL. Red lines indicate normalized mean MFIs of isotype-treated cultures. **f** Quantification of viable splenic T-ALL cells 6–7 days after co-culture with enriched tumor-associated myeloid cells +/- exogenous IGF1 (100 ng/ml) +/- anti-ICAM-1 and anti-VCAM-1 blocking antibodies (10 µg/ml each). Results were normalized to T-ALL-myeloid co-cultures with isotype control antibodies. Bars represent mean + SEM from *n* = 5 independent experiments with distinct color-coded primary T-ALL; symbols represent averages of 2-3 technical replicate wells. The red line indicates the normalized mean viability of isotype-treated co-cultures. Statistical significance was determined by (**a**, **b**, **c**, **f**) a two-sided repeated measures one-way ANOVA with the Holm-Sidak correction, and (**e**) two-sided paired Student *t*-tests; *P*-values: *<0.05, **<0.01, ***<0.001. ns, not significant. Source data are provided as a Source Data file.

Next, we tested whether interactions of the candidate integrins LFA-1 or ITGβ1 with the corresponding adhesion molecules ICAM-1 and VCAM-1, respectively, are required for myeloid-mediated T-ALL support. LN3 T-ALL cells were co-cultured with tumor-associated myeloid cells in the presence of anti-ICAM-1 and/or anti-VCAM-1 blocking antibodies. Blocking either ICAM-1 or VCAM-1 suppressed T-ALL survival. Notably, blocking both ICAM-1 and VCAM-1 further decreased survival to levels comparable to T-ALL cells cultured alone (Fig. 2a, Supplementary Fig. 5a, b), indicating that these adhesion molecules are essential for myeloid-mediated T-ALL support. Blocking antibodies against the corresponding integrins ITGαL and ITGβ1 (Fig. 2b), as well as a small molecule inhibitor of LFA-1 (Fig. 2c), also significantly diminished T-ALL survival. Isotype-matched antibodies that bound T-ALL cells did not alter T-ALL viability in co-cultures with myeloid cells (Supplementary Fig. 5c, d), demonstrating that blocking antibodies against integrins did not reduce T-ALL survival by inducing Fc receptor-mediated phagocytosis of opsonized T-ALL cells. These results demonstrate that tumor-associated myeloid cells support T-ALL growth through integrin-mediated close contact in vitro.

We previously found that tumor-associated myeloid cells support T-ALL growth by activating IGF1R signaling^14^. Integrin and IGF1R signaling pathways are interconnected^23,28^. Thus, we hypothesized that inhibiting integrin adhesion would diminish myeloid-mediated IGF1R activation in T-ALL cells. Indeed, neutralizing antibodies against ICAM-1 and VCAM-1 significantly diminished the levels of activated phosphorylated (p)IGF1R and the downstream kinase pAKT (Fig. 2d, e). Blocking both ICAM-1 and VCAM-1 did not reduce IGF1R or AKT activation in T-ALL cells more than blocking either adhesion molecule individually (Supplementary Fig. 6a, b), despite more significantly reducing T-ALL survival (Fig. 2a). Thus, in addition to activating IGF1R, other mechanisms must contribute to the pro-survival effects of integrin signaling in T-ALL. Because blocking both ICAM-1 and VCAM-1 suppressed T-ALL survival most significantly, we leveraged this combination treatment for additional in vitro studies. Supplying exogenous IGF1 enhances survival of T-ALL cells co-cultured with tumor-associated myeloid cells^14^. To determine whether blockade of integrin-mediated physical interactions would prevent sensitization of T-ALL cells to IGF1, we co-cultured T-ALL cells with myeloid cells in the presence or absence of exogenous IGF1 and/or antibodies against adhesion molecules. Consistent with our previous findings, addition of IGF1 increased viability of T-ALL cells only in the presence of tumor-associated myeloid cells (Fig. 2f). Notably, blockade of ICAM-1 and VCAM-1 significantly reduced myeloid-mediated sensitization of T-ALL cells to IGF1, demonstrating that integrin activation is required for the full responsiveness of T-ALL cells to IGF1. Taken together, integrin-mediated close contact between T-ALL cells and tumor-associated myeloid cells plays an essential role in promoting the survival of T-ALL, in part by enabling activation of IGF1R signaling.

### ICAM-1- and VCAM-1-mediated cell adhesion is critical for T-ALL establishment and/or progression in vivo

**Fig. 3:**
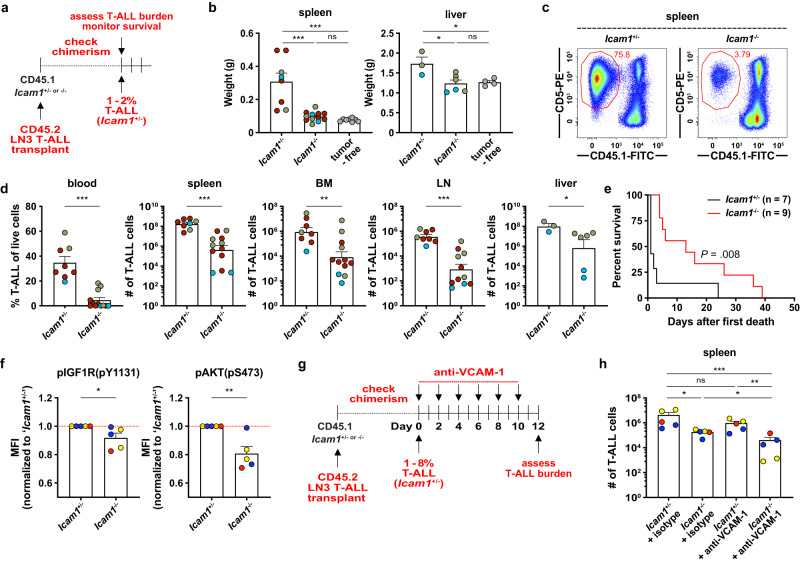
ICAM-1- and VCAM-1-mediated cell adhesion is critical for T-ALL establishment and/or progression in vivo. **a** Schematic of experiment to test if T-ALL burden or mouse survival are impacted by *Icam1* deficiency. **b** Quantification of splenic and liver weights of *Icam1*^+/-^ and *Icam1*^-/-^ mice transplanted with primary LN3 T-ALL or tumor-free littermates. Results were compiled from *n* = 2 (liver) or 3 (spleen) experiments, each with a distinct color-coded primary T-ALL; symbols represent individual mice. **c** Representative flow cytometry plots showing diminished splenic T-ALL burden in LN3 T-ALL-bearing *Icam1*^-/-^ versus *Icam1*^+/-^ littermates. **d** Quantification of the frequency of circulating T-ALL blasts or the number of T-ALL cells in spleen, bone marrow (BM), inguinal lymph nodes (LN), and liver in *Icam1*^+/-^ and *Icam1*^-/-^ mice from the experiments in (**b**). **e** Cumulative survival of *Icam1*^+/-^ and *Icam1*^-/-^ leukemic mice from *n* = 3 independent experiments, each with a different primary LN3 T-ALL, using the strategy in (**a**). Kaplan–Meier survival curves were normalized to the first day of death in each experiment. **f** Quantification of pIGF1R and pAKT expression in splenic T-ALL cells from *Icam1*^+/-^ and *Icam1*^-/-^ mice treated with an isotype control antibody from the experiments in Supplementary Fig. 7b, c. Results were normalized to the MFI of *Icam1*^+/-^ mice within each experiment and were compiled from *n* = 3 independent experiments, each with a distinct color-coded primary T-ALL; symbols represent individual mice. Red lines indicate normalized mean MFIs from *Icam1*^+/-^ mice. **g** Experimental schematic depicting dosing schedule for anti-VCAM-1 or isotype control antibodies following establishment of LN3 T-ALL (1–8% spleen chimerism) in *Icam1*^-/-^ and *Icam1*^+/-^ mice. Leukemia burden was assessed 2 days after the final antibody injection. **h** Quantification of T-ALL cells in the spleens of *Icam1*^+/-^ and *Icam1*^-/-^ mice treated with either anti-VCAM-1 or isotype control antibodies. Results were compiled from *n* = 3 experiments, each with a distinct color-coded primary T-ALL; symbols represent individual mice. Throughout this figure, bars depict means + SEM of cumulative data. Statistical significance was determined by (**b**, **h**) a two-sided repeated measures one-way ANOVA with the Holm-Sidak correction, (**d**, **f**) two-sided unpaired Student *t*-tests, and (**e**) log-rank tests; *P*-values: *<0.05, **<0.01, ***<0.001. ns, not significant. Source data are provided as a Source Data file.

To determine whether integrin-mediated adhesion promotes T-ALL establishment or progression in vivo, we engrafted primary LN3 T-ALL cells into *Icam1*^-/-^ mice and *Icam1*^+/-^ littermate controls and assessed leukemic burden after T-ALL establishment in control mice (1-2% blood chimerism; Fig. 3a). In *Icam1*^*-/-*^ mice, spleen and liver weights were reduced to levels comparable to tumor-free littermate *Icam1*^*+/-*^ controls (Fig. 3b). Moreover, circulating T-ALL blasts were significantly reduced, as was T-ALL burden in multiple organs including the spleen, bone marrow (BM), lymph nodes (LN), and liver (Fig. 3c, d). In keeping with these findings, *Icam1* deficiency prolonged survival following transplantation of T-ALL (Fig. 3e). To obtain a sufficient number of littermate controls for these experiments, we compared T-ALL burden and survival in *Icam1*^*-/-*^ versus *Icam1*^*+/-*^ mice. We found that haploinsufficiency of *Icam1* results in slightly diminished expression of ICAM-1 on myeloid cells relative to wild-type *Icam1*^*+/+*^ cells (Supplementary Fig. 7a). Thus, the reduced T-ALL burden and increased survival in *Icam1*^*-/-*^ versus *Icam1*^*+/-*^ mice would, if anything, underestimate the role of ICAM-1 in supporting T-ALL survival and progression. To test the possibility that ICAM-1 induces survival signals in T-ALL cells in vivo, we compared pIGF1R and pAKT levels in T-ALL cells from *Icam1*^*+/-*^ versus *Icam1*^*-/-*^ mice. Consistent with results from co-culture assays, *Icam1* deficiency significantly diminished IGF1R and AKT activation (Fig. 3f). To determine whether VCAM-1 blockade would further decrease T-ALL growth and progression in vivo, we transplanted primary LN3 T-ALL cells into *Icam1*^-/-^ mice and *Icam1*^+/-^ littermate controls and administered an anti-VCAM-1 blocking antibody or a relevant isotype control antibody after T-ALL establishment (>1% spleen chimerism; Fig. 3g). Notably, treatment of *Icam1*^-/-^ mice with anti-VCAM-1 reduced leukemic burden significantly more than single *Icam1* deficiency or anti-VCAM-1 treatment (Fig. 3h), indicating that both ICAM-1 and VCAM-1 contribute to T-ALL progression. Consistent with in vitro findings, despite the reduction in T-ALL burden, anti-VCAM-1 blocking antibodies did not reduce IGF1R or AKT activation in T-ALL cells from *Icam1*^-/-^ mice (Supplementary Fig. 7b, c), suggesting ICAM-1 and VCAM-1-mediated integrin activation promote T-ALL survival through additional mechanisms beyond IGF1R activation in vivo. Together, these results indicate that ICAM-1- and VCAM-1-mediated integrin activation are critical for T-ALL establishment and/or progression.

### ICAM-1- and VCAM-1-mediated cell adhesion are critical for survival and progression of established T-ALL in vivo

**Fig. 4:**
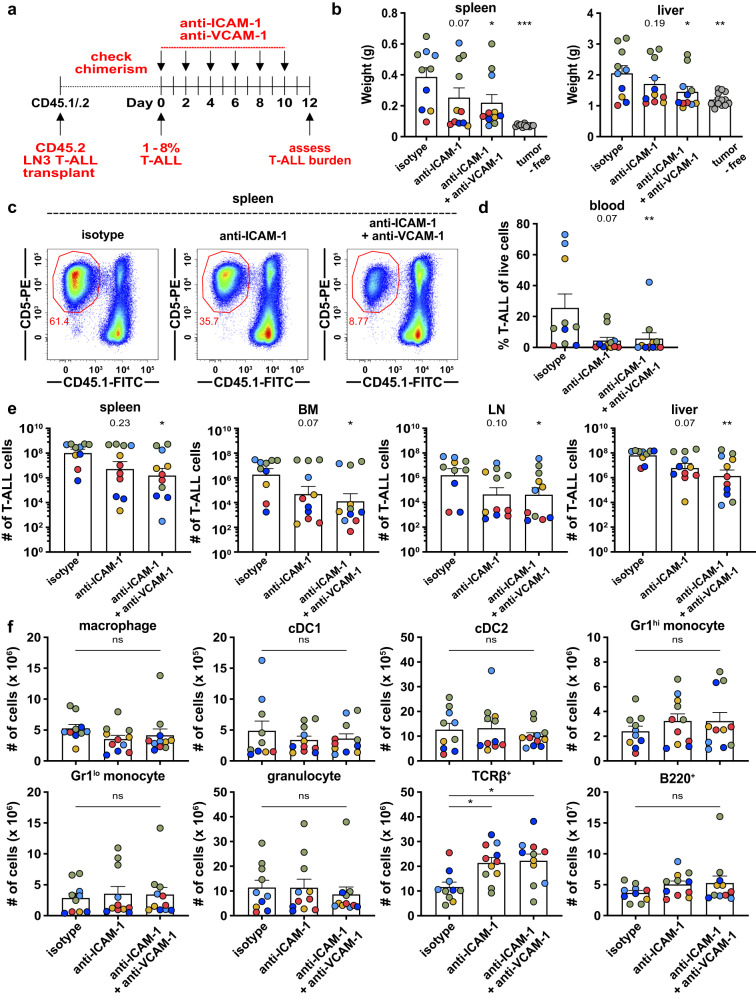
Inhibition of ICAM-1- and VCAM-1-mediated cell adhesion reduces T-ALL burden in vivo. **a** Experimental schematic depicting dosing schedule for anti-ICAM-1, anti-VCAM-1, or isotype control antibodies following establishment of LN3 T-ALL ( > 1% spleen chimerism) in congenic hosts. Leukemia burden was assessed 2 days after the final antibody injection. **b** Quantification of spleen and liver weights from the experiments depicted in (**a**). Tumor-free, age-matched mice were included for comparison. Bars depict the mean + SEM of cumulative data from *n* = 5 experiments, each with a distinct color-coded primary T-ALL; symbols represent individual mice. **c** Representative flow cytometry plots showing a decrease in T-ALL burden in the spleens of LN3 T-ALL-transplanted mice following treatment with anti-ICAM-1 +/- anti-VCAM-1 antibodies (100 µg each per mouse). **d**, **e** Quantification of the (**d**) frequency of circulating T-ALL blasts in the blood and (**e**) number of T-ALL cells in the spleen, BM, LN, and liver from the same experiments as in (**b**). **f** Quantification of the indicated myeloid subsets or TCRβ^+^ or B220^+^ host cells in the spleens of mice from the same experiments as in (**d**, **e**). Statistical significance was determined by (**b**, **d**, **e**, **f**) a two-sided repeated measures one-way ANOVA with the Holm-Sidak correction; *P*-values: *<0.05, **<0.01, ***<0.001. ns, not significant. Source data are provided as a Source Data file.

To evaluate the contribution of integrin-mediated cell adhesion to T-ALL progression after tumor establishment, we administered an anti-ICAM-1 blocking antibody alone or in combination with an anti-VCAM-1 blocking antibody after LN3 leukemic burden reached 1–8% in the spleen (Fig. 4a). Consistent with in vitro results, combination treatment with anti-ICAM-1 and anti-VCAM-1 antibodies significantly reduced spleen and liver weights (Fig. 4b). Circulating T-ALL blasts and T-ALL burden were also reduced by one to two orders of magnitude in the spleen, BM, LN, and liver (Fig. 4c–e). A similar trend was observed for mice treated with anti-ICAM-1 antibody alone (Fig. 4c–e). Notably, these antibodies did not deplete myeloid cells in leukemic mice (Fig. 4f, Supplementary Fig. 7d, e), indicating that the observed reduction in T-ALL burden resulted from inhibition of cell adhesion, rather than from depletion of leukemia-supportive myeloid cells. Collectively, ICAM-1- and VCAM-1-mediated cell adhesion play a critical role in promoting T-ALL growth and progression in vivo.

### FAK and PYK2 are activated in T-ALL in an ICAM-dependent manner and support leukemia survival in part by activating IGF1R signaling

**Fig. 5:**
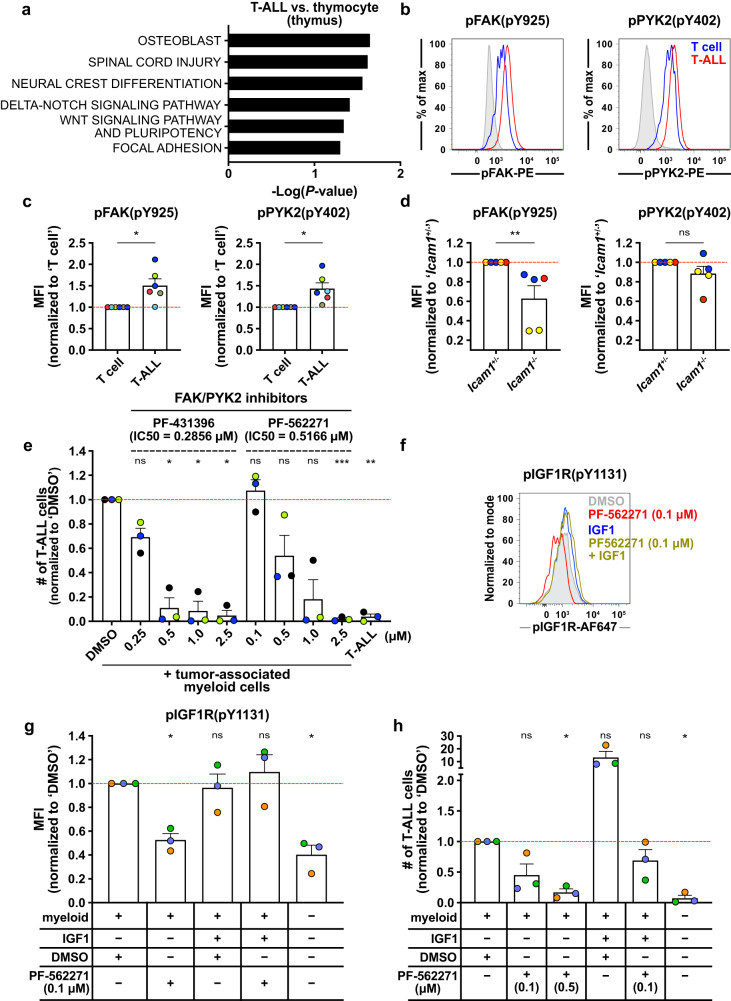
Inhibition of FAK and PYK2 signaling reduces T-ALL survival in vitro. **a** The top 6 pathways upregulated in thymic T-ALL cells versus healthy thymocytes identified using Enrichr with WikiPathways 2019 Mouse gene sets. **b**, **c** (**b)** Representative flow cytometry plots and (**c**) quantification of pFAK and pPYK2 levels in T-ALL (CD45.2^+^CD5^+^) relative to host T cells (CD45.1^+^CD5^+^) in the same leukemic spleens. Isotype control stain in gray. Results were normalized to mean MFIs of T cells and compiled from *n* = 6 independent experiments. **d** Quantification of pFAK and pPYK2 levels in splenic LN3 T-ALL from *Icam1*^+/-^ and *Icam1*^-/-^ hosts treated with an isotype control antibody from experiments in Supplementary Fig. 7b, c. Results were normalized to the MFI of *Icam1*^+/-^ mice and compiled from *n* = 3 independent experiments; symbols represent individual mice. **e** Quantification of viable T-ALL cells in myeloid co-cultures treated 4 days after culture initiation with FAK/PYK2 dual inhibitors (PF-431396 or PF-562271) and quantified 2–3 days later. Results were normalized to DMSO treated control cultures and compiled from *n* = 3 independent experiments; symbols represent averages of 2–3 replicate wells. **f**, **g** (**f**) Representative flow cytometry plots and (**g**) quantification of pIGF1R levels in LN3 T-ALL cells co-cultured with myeloid cells and PF-562271 (0.1 μM) and/or IGF1 (100 ng/ml), or DMSO for 4–5 days. Results were normalized to DMSO-treated cultures and compiled from *n* = 3 independent experiments; symbols represent averages of 2-3 technical replicate wells. **h** Quantification of viable splenic T-ALL cells 4–5 days after co-culture with enriched myeloid cells +/- exogenous IGF1 (100 ng/ml) and/or PF-562271 (0.1 or 0.5 μM, as indicated). Results were normalized to T-ALL cells co-cultured with myeloid cells + DMSO alone and compiled from *n* = 3 independent experiments; symbols represent the average of 2-3 technical replicate wells per experiment. Bars represent means + SEM; red lines indicate normalized means of controls. Distinct primary T-ALLs are color-coded. Statistical significance was determined by (**a**) Fisher exact tests, (**c**) two-sided paired Student *t*-tests, (**d**) two-sided unpaired Student *t*-tests, and (**e**, **g**, **h**) a two-sided repeated measures one-way ANOVA with the Holm-Sidak correction: *<0.05, **<0.01, ***<0.001. ns, not significant. Source data are provided as a Source Data file.

Blocking multiple integrins and/or adhesion molecules simultaneously could be therapeutically challenging^36,37^; thus, we sought a common targetable signal downstream of integrin activation. Our transcriptional profiling data from thymic T-ALL vs healthy thymocytes revealed that the “focal adhesion” pathway was among the top pathways preferentially activated in T-ALL cells (*P* = 0.049; Fig. 5a). FAK and PYK2 are two integrin-associated kinases in the focal adhesion pathway that have been shown to play important roles in tumor progression^38,39^. Thus, we hypothesized that these kinases would be activated by myeloid-mediated integrin signaling in T-ALL cells and would contribute to leukemia growth. Indeed, FAK phosphorylation at Y397 and Y925, but not at Y861, and PYK2 phosphorylation at Y402 were significantly elevated in T-ALL cells relative to T cells from leukemic spleens (Fig. 5b, c, Supplementary Fig. 8a, b). Activation of FAK and PYK2 are multi-step processes, in which initial auto-phosphorylation at FAK Y397 and at PYK2 Y402 recruit Src-family kinases that contribute to full kinase activation via subsequent phosphorylation at residues including FAK Y925, leading to survival signaling through the Ras/MAPK pathway^40,41^. pFAK and pPYK2 levels were also elevated in T-ALL cells relative to healthy thymocytes (Supplementary Fig. 8c, d). Notably, pFAK levels in T-ALL cells were significantly diminished in *Icam1*^-/-^ mice relative to *Icam1*^+/-^ littermate controls, with a similar, but non-significant, trend for pPYK2 (Fig. 5d), demonstrating that activation of FAK and PYK2 in T-ALL cells is dependent on ICAM-1 expression in the leukemic microenvironment. Furthermore, blocking antibodies to ICAM-1 and VCAM-1 in co-cultures of T-ALL and myeloid cells reduced the frequency of T-ALL cells with activated FAK and PYK2 (Supplementary Fig. 8e, f), confirming a link between integrin signaling and FAK/PYK2 activation in T-ALL cells upon ICAM-1 and VCAM-1 dependent interactions with myeloid cells.

We next tested whether activation of FAK and PYK2 is required for myeloid-mediated support of T-ALL. In co-cultures, small molecule inhibitors of FAK/PYK2 significantly reduced LN3 T-ALL survival in a concentration dependent manner (Fig. 5e). Because integrin signaling promotes IGF1R activation in T-ALL cells (Fig. 2d, e and Fig. 3f), we tested whether FAK and PYK2 were required for integrin-mediated IGF1R activation and sensitization to exogenous IGF1. Indeed, inhibiting FAK/PYK2 significantly decreased myeloid-mediated activation of IGF1R in T-ALL cells (Fig. 5f, g) and diminished the enhanced survival of T-ALL to exogenous IGF1 in myeloid co-cultures (Fig. 5h). However, in the presence of exogenous IGF1, inhibition of FAK/PYK2 did not reduce IGF1R phosphorylation (Fig. 5f, g), even though viable T-ALL cell numbers decreased significantly (Fig. 5h). These findings indicate that FAK and PYK2 activation support T-ALL survival through additional mechanisms beyond IGF1R phosphorylation. Collectively, FAK and PYK2 kinases play a critical role in myeloid-mediated T-ALL support downstream of integrin signaling and contribute to IGF1R sensitization of T-ALL in the presence of myeloid cells.

### FAK and PYK2 are critical for T-ALL survival and progression in vivo

**Fig. 6:**
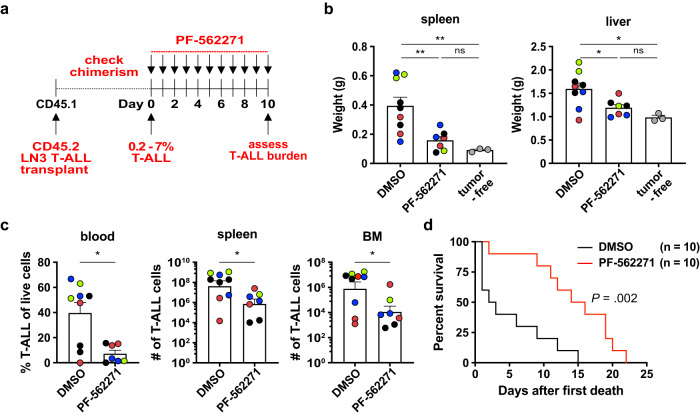
FAK and PYK2 are critical for T-ALL survival and progression in vivo. **a** Experimental schematic depicting dosing schedule for PF-562271 or DMSO vehicle control treatment to inhibit FAK and PYK2 signaling in mice following establishment of LN3 T-ALL in congenic recipients (>0.2% spleen chimerism). **b** Quantification of spleen and liver weights from mice transplanted with primary LN3 T-ALL after treatment with PF-562271 or DMSO, as in (**a**). Tumor-free littermates were included for comparison. **c** Quantification of the frequency of circulating T-ALL blasts and the number of T-ALL cells in the spleen and BM from the same experiments as in (**b**). Bars depict the mean + SEM of cumulative data from *n* = 4 experiments, each with a distinct color-coded primary T-ALL; symbols represent individual mice. **d** Graph shows cumulative survival of PF-562271 or DMSO-treated leukemic mice from *n* = 3 independent experiments, each with a different primary LN3 T-ALL, using the strategy as in (**a**). Kaplan–Meier survival curves were normalized to the first day of death in each experiment. Statistical significance was determined by (**b**) a two-sided repeated measures one-way ANOVA with the Holm-Sidak correction, (**c**) two-sided unpaired Student *t*-tests, and (**d**) log-rank tests; *P*-values: *<0.05, **<0.01. ns not significant. Source data are provided as a Source Data file.

To assess the contribution of FAK/PYK2 to T-ALL progression in vivo, we treated leukemic mice with PF-562271 (Fig. 6a). Inhibition of FAK and PYK2 diminished spleen and liver weights to those of healthy littermate controls (Fig. 6b). FAK/PYK2 inhibitor treatment also significantly reduced circulating T-ALL blasts and tumor burden by one to two orders of magnitude in the spleen and BM (Fig. 6c, Supplementary Fig. 8g). Furthermore, in vivo administration of this FAK/PYK2 inhibitor significantly prolonged survival of mice with established LN3 T-ALL (Fig. 6d). PF-562271 altered not only the T-ALL cells, but also the TME, consistent with previous findings^42^. The number of cDC1, cDC2, and Gr1^lo^ monocytes was reduced, but the overall number of myeloid cells and the number of macrophages did not significantly decline (Supplementary Fig. 8h, i). Given that macrophages in the splenic and hepatic TME have the most potent capacity among myeloid subsets to support T-ALL^14^, the observed reduction of leukemic burden and prolonged survival following FAK/PYK2 inhibitor treatment likely resulted mainly from reduced FAK/PYK2 signaling in T-ALL cells, rather than altered myeloid composition; however, we cannot rule out that these inhibitors impact other cell types expressing FAK/PYK2, such as leukemia-supportive myeloid cells, thus indirectly reducing T-ALL burden. In fact, the ability of FAK/PYK2 inhibitors to diminish leukemia-supportive myeloid cells as well as integrin signaling in T-ALL cells could be an added benefit of targeting this pathway.

To determine whether integrin-mediated myeloid support of T-ALL was relevant in a distinct T-ALL mouse model, we carried out comparable experiments in human CD2 promoter-driven LIM domain only 2 (LMO2) transgenic mice^43^. As in LN3 T-ALL, LMO2 T-ALL cells expressed higher levels of *Itgα9* and *Itgβ1* transcripts relative to healthy thymocytes and higher levels of ITGα9, ITGβ1, and ITGβ2 protein relative to non-leukemic T cells from the same splenic tumor environment (Supplementary Fig. 9a, b). In addition, integrin-mediated cell adhesion promoted survival of LMO2 T-ALL cells co-cultured with tumor-associated myeloid cells (Supplementary Fig. 9c). Moreover, inhibition of FAK/PYK2 significantly reduced survival of T-ALL cells co-cultured with tumor-associated myeloid cells (Supplementary Fig. 9d). Taken together, these concordant findings in multiple mouse models of T-ALL demonstrate that tumor-associated myeloid cells promote T-ALL survival and growth via activation of integrin signaling and downstream FAK/PYK2 kinases.

### Integrin activation by myeloid cells promotes primary patient T-ALL survival and is associated with inferior outcomes

**Fig. 7:**
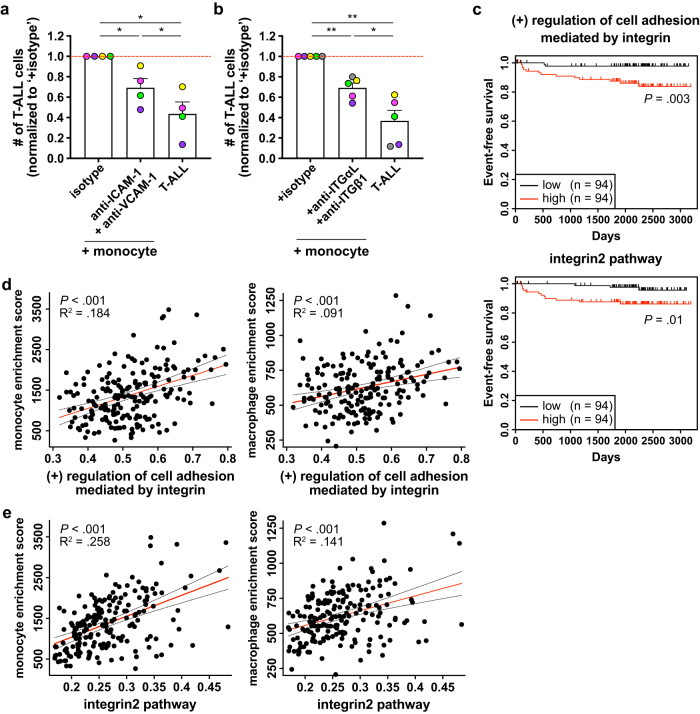
Human myeloid cells promote survival of primary patient T-ALL cells in an integrin-dependent manner in vitro, and enriched gene signatures of integrin pathways in patients are associated with worse prognosis. **a**, **b** Quantification of viable primary patient T-ALL cells cultured for 6–7 days alone or with monocytes from healthy donor peripheral blood mononuclear cells (PBMCs). Co-cultures were carried out in the presence of (**a**) anti-ICAM-1 (20 µg/ml) and anti-VCAM-1 (10 µg/ml) blocking antibodies, (**b**) anti-integrin αL (ITGαL) and anti-ITGβ1 blocking antibodies (10 µg/ml for each), or isotype control antibodies as indicated. Results were normalized to isotype-treated cultures in each experiment. Bars represent means + SEM from *n* = 2–3 independent experiments using 4–5 distinct, color-coded patient-derived T-ALLs; symbols represent the average of 2 technical replicate wells. Red lines indicate the normalized mean T-ALL viability in isotype-treated cultures. **c** Longitudinal event-free survival is plotted for pediatric T-ALL patients stratified into two equal groups based on their gene signature enrichment scores for “(+) regulation of cell adhesion mediated by integrin” (top) or “integrin2 pathway” (bottom). Patient data were analyzed from published datasets from 264 T-ALL patients from the TARGET ALL Phase II trial^12^. **d**, **e** Plots depict correlations between enrichment scores of the gene signatures (**d**) “(+) regulation of cell adhesion mediated by integrin” or (**e**) “integrin2 pathway” with enrichment scores for monocytes (left) and macrophages (right) in patient samples. The red and black dotted lines represent the best-fit line and 95% confidence bands, respectively. Symbols represent each patient. Statistical significance was determined by (**a**, **b**) a two-sided repeated measures one-way ANOVA with the Holm-Sidak correction, (**c**) log-rank tests, and (**d**, **e**) simple linear regression analyses; *P*-values: *<0.05, **<0.01. ns not significant. Source data are provided as a Source Data file.

**Fig. 8:**
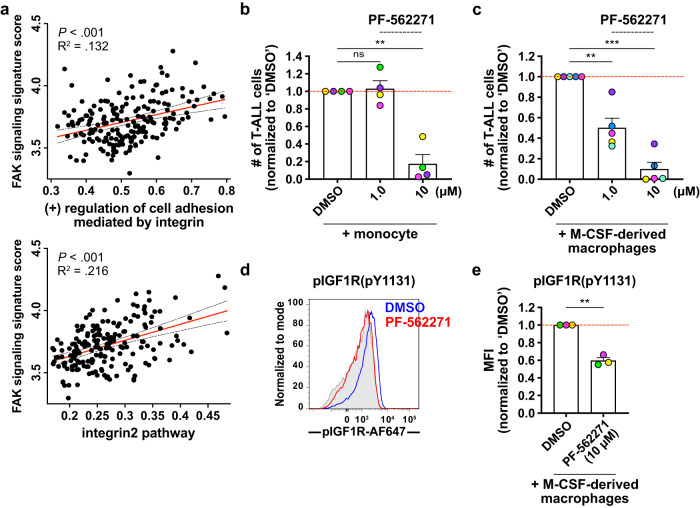
FAK/PYK2 signaling is critical for primary patient T-ALL survival in vitro. **a** Plots show the correlation between enrichment scores for the gene signatures “(+) regulation of cell adhesion mediated by integrin” (left) or “integrin2 pathway” (right) with signature scores of FAK signaling in patient samples from the TARGET ALL Phase II trial^12^. The enrichment scores for “FAK signaling signature” were calculated using the gene signatures from a prior study^77^. The red and black dotted lines represent the best-fit line and 95% confidence bands, respectively. Symbols represent each patient. **b**, **c** Quantification of viable primary patient T-ALL cells co-cultured for 4 days with (**b**) PBMC-derived monocytes or (**c**) M-CSF-derived macrophages 2–3 days after addition of the indicated concentrations of PF-562271 or vehicle control (DMSO). Results were normalized to DMSO-treated cultures for each experiment. Bars represent means + SEM from *n* = 3 independent experiments using 4-5 distinct, color-coded patient-derived T-ALLs; symbols represent the average of 2 technical replicate wells. Red lines indicate the normalized mean T-ALL viability in DMSO-treated cultures. **d** Representative flow cytometry plots of pIGF1R in primary patient T-ALL cells co-cultured with M-CSF-derived macrophages for 4 days before addition of 10 μM of PF-562271 (red) or vehicle control (DMSO; blue). pIGF1R levels were assessed after 1–2 days. Isotype control stains are shaded in gray. **e** Quantification of pIGF1R MFI levels in co-cultured T-ALL cells, as in (**d**). Results were normalized to the MFI of DMSO-treated cultures within each experiment. Bars represent mean + SEM from *n* = 3 independent experiments, each with a distinct color-coded primary T-ALL; symbols represent the average of 2–3 technical replicate wells. The red line indicates the normalized mean MFI of DMSO-treated cultures. Statistical significance was determined by (**a**) simple linear regression analyses, (**b**, **c**) a two-sided repeated measures one-way ANOVA with the Holm-Sidak correction, and (**e**) two-sided paired Student *t*-tests; *P*-values: **<0.01, ***<0.001. ns not significant. Source data are provided as a Source Data file.

We previously demonstrated that human peripheral blood mononuclear cell (PBMC)-derived myeloid cells support survival of primary patient T-ALL cells^14^, and thus tested whether integrin-mediated cell adhesion was also important for myeloid-mediated survival of human T-ALL. We first assessed expression of integrins and cell adhesion ligands by T-ALL cells and PBMC-derived myeloid cells, respectively. Consistent with mouse models, VLA-4, integrin α9β1, and LFA-1 were expressed by patient T-ALL cells (Supplementary Fig. 10a). ICAM-1 was highly expressed by both monocytes and M-CSF-derived macrophages, but VCAM-1 expression was negligible (Supplementary Fig. 10b, c). To determine the impact of integrin-mediated cell adhesion on myeloid support of T-ALL, primary pediatric T-ALL cells were co-cultured with monocytes or M-CSF-derived macrophages in the presence or absence of blocking antibodies against ICAM-1 and VCAM-1. Blocking adhesion molecules or integrins significantly diminished survival of T-ALL cells co-cultured with monocytes (Fig. 7a, b), but not with M-CSF-derived macrophages (Supplementary Fig. 10d). Isotype-matched antibodies that bound T-ALL cells did not reduce T-ALL survival in myeloid co-cultures (Supplementary Fig. 10e, f), indicating that Fc receptor-mediated phagocytosis of opsonized T-ALL cells was not a significant factor in reduced T-ALL viability after treatment anti-integrin antibodies. To test whether integrin signaling is associated with altered patient outcomes, we analyzed published datasets from pediatric T-ALL patients^12^ and found that increased gene signatures of the “(+) regulation of cell adhesion mediated by integrin” and “integrin2” pathways correlate significantly with diminished event-free survival (Fig. 7c). Also, elevated expression of integrin pathway gene signatures correlates significantly with monocyte and macrophage enrichment scores in T-ALL patient samples (Fig. 7d, e), suggesting myeloid cells activate integrin signaling in patient T-ALL cells. Furthermore, integrin and FAK pathway signature scores correlate significantly with one another in patient T-ALL samples (Fig. 8a), concordant with a link between integrin and FAK signaling in T-ALL patients. Notably, survival of T-ALL cells co-cultured with monocytes or M-CSF-derived macrophages was significantly impaired in the presence of the FAK/PYK2 inhibitor PF-562271 (Fig. 8b, c), as was activation of IGF1R in T-ALL cells (Fig. 8d, e). Collectively, these results indicate that myeloid cells support survival of patient T-ALL cells via integrin-mediated cell adhesion and downstream activation of FAK/PYK2, which is required for IGF1R activation.

## Discussion

Although tumor-associated myeloid cells promote T-ALL progression by activating IGF1R signaling^13,14^, which is required for initiation and growth of T-ALL^44^, exogenous IGF1 alone is not sufficient to support T-ALL survival^14^. Notably, the presence of tumor-associated myeloid cells enhances the response of T-ALL cells to exogenous IGF1^14^. These findings suggest that myeloid cells employ mechanisms in addition to IGF1 production to promote T-ALL survival in the TME. Analysis of our published RNA-seq datasets^14^ revealed that gene expression signatures of cell-cell interactions were significantly enriched in thymic T-ALL cells relative to healthy thymocytes, consistent with our previous imaging results showing that T-ALL cells make prolonged and frequent contacts with DCs in the thymus^13^. Based on these findings, we hypothesized that physical interactions are critical for myeloid-mediated T-ALL support. Here we find that T-ALL cells express elevated levels of integrins known to be involved in T cell-myeloid interactions^22^, and leukemia-associated myeloid cells express elevated levels of cell adhesion molecules that bind and activate integrins. Moreover, inhibition of integrin-mediated cell adhesion results in a significant reduction in T-ALL cellularity both in vitro and in vivo and prolongs survival in a mouse model of T-ALL. In other cancers, integrins have been shown to interact with IGF1R to promote signaling that results in tumor survival, migration, and therapeutic resistance^28,45,46^. Notably, we found that blocking integrin-mediated cell adhesion diminishes IGF1R and AKT activation in T-ALL cells co-cultured with tumor-associated myeloid cells. Inhibition of integrin adhesion also impairs myeloid-mediated sensitization of T-ALL cells to exogenous IGF1. These findings link the dual requirements of integrin activation and IGF1R signaling for myeloid-mediated support of T-ALL survival. Consistent with mouse models, survival of primary patient T-ALL cells co-cultured with monocytes was significantly reduced by blocking integrin-mediated cell adhesion. Also, pediatric T-ALL patients with elevated expression of integrin pathway genes have increased gene signatures associated with myeloid cells and inferior outcomes. Altogether, these results indicate that myeloid cells promote T-ALL survival through a combination of close contact with leukemia cells, driven by integrin-mediated interactions and IGF1R activation.

In keeping with a critical role for integrin signaling in supporting T-ALL survival, FAK/PYK2 signaling contributes to myeloid-mediated T-ALL support. FAK and PYK2 promote tumor initiation, growth, and invasion across multiple cancer types including hematologic malignancies^38,39,47–49^. For example, a prior study revealed that FAK supports survival of acute myeloid leukemia (AML) cells by promoting leukemia-stroma interactions^38^. In addition, FAK has been reported to promote survival of *PTEN*-null T-ALL by activating survival signals that compensate for reduced PI3K-AKT activtiy^47^. However, mechanisms underlying activation of FAK/PYK2 in T-ALL, and the downstream consequences have remained largely unresolved. Prior studies revealed that FAK and PYK2 are activated by integrin signaling following cell adhesion to a number of integrin ligands, including adhesion molecules like ICAM-1 and VCAM-1^22^. FAK activation is known to be a multi-step process including initial auto-phosphorylation of the Y397 residue, followed by the phosphorylation of Y925, leading to activation of survival signaling pathways, including the ERK2/MAP kinase cadcase^40,50^. As for PYK2, the Y402 site is phosphorylated in a Src dependent manner in response to cell adhesion via integrins^41^. We find that FAK and PYK2 are highly activated at these phosphorylation sites in T-ALL cells relative to T cells from the same leukemic environment, consistent with elevated integrin signaling in T-ALL. Notably, we found that multiple myeloid subsets from the leukemic microenvironment express elevated levels of ICAM-1 and VCAM-1, suggesting that integrin binding to these adhesion molecules could activate FAK/PYK2 in T-ALL. Given that FAK and PYK2 are activated downstream of integrin signaling and promote T-cell proliferation and survival^24^, we hypothesized that myeloid-mediated T-ALL support would be dependent on FAK and/or PYK2 signaling. Indeed, pharmacologic inhibition of these kinases impaired myeloid-mediated survival of mouse and patient T-ALL survival in a dose-dependent manner in vitro, in part by diminishing IGF1R activation. FAK/PYK2 inhibition also reduced T-ALL burden in vivo and prolonged survival of leukemic mice, pointing to FAK/PYK2 as potential therapeutic targets for T-ALL.

FAK and PYK2 can be activated not only by integrins, but also by soluble factors, including vascular endothelial growth factor (VEGF), the chemokine CCL19, and interleukin-1β (IL-1b)^51–53^. When mouse or patient T-ALL cells were co-cultured with tumor-associated or PBMC-derived myeloid cells, respectively, they exhibited fairly uniform sensitivity to FAK/PYK2 dual inhibitors, whereas the efficacy of inhibiting integrin-mediated cell adhesion varied between distinct primary T-ALLs. These findings suggest that tumor-associated myeloid cells may activate FAK and PYK2 in T-ALL cells not only through integrin signaling, but also through additional pathways. A previous study reported that *Vegfa* and *Il1b* transcripts are elevated in leukemia-associated macrophages in a NOTCH1-induced model of T-ALL^54^, suggesting secreted myeloid factors could promote FAK/PYK2 activation in T-ALL cells. Thus, a better understanding of the mechanisms by which tumor-associated myeloid cells activate FAK and PYK2 signaling and how these pathways contribute to T-ALL progression could reveal other therapeutic opportunities in T-ALL.

Although inhibiting integrin adhesion or FAK/PYK2 signaling significantly diminished leukemia burden and prolonged survival in a mouse model of T-ALL, the mice eventually succumbed to leukemia, suggesting other cell-intrinsic pathways or supportive elements of the TME would need to be inhibited simultaneously for therapeutic intervention. Previous studies have identified other signals in the TME that support T-ALL. For example, despite activating mutations in *NOTCH1*, T-ALL cells require NOTCH ligands, which are expressed by non-hematopoietic stromal cells in a variety of organs^25,55^, to activate NOTCH1^9,44,56^. IL-7, which is produced by TECs, has also been implicated in promoting T-ALL progression^19,20^. IGF1 is produced in the liver as a growth factor but interestingly, we have found it is also expressed by leukemia-associated myeloid cells, and IGF1R signaling is critical for T-ALL initiation and growth^13,14,44^. Furthermore, CXCL12 expression by BM stromal cells has been shown to promote T-ALL initiation and progression^17,18^. Cell adhesion molecules such as integrins and CD44 have also been implicated in T-ALL pathogenesis through interactions with BM stromal cells^57–59^. Thus, as our study reveals a critical role for integrins in myeloid-mediated support of T-ALL, it will likely be important to target signals provided by hematopoietic and other stromal cells in the TME to improve therapeutic strategies for T-ALL.

Collectively, this study demonstrates that tumor-associated myeloid cells support T-ALL through integrin-mediated close contact. Inhibition of integrins or downstream FAK/PYK2 signaling delays T-ALL progression and improves survival of leukemic mice. Integrins are associated with T-cell localization to and migration within secondary lymphoid organs, such as LNs where pro-leukemic cytokines like IL-7 are produced^20,60^. Moreover, integrin-mediated migration and adhesion is activated by chemokine receptors like CXCR4, which promotes T-ALL initiation and maintenance in the BM^17,18,61^. Thus, inhibition of integrin-mediated physical interactions could target multiple mechanisms by which the TME supports T-ALL. It will be important to evaluate whether targeting integrins or downstream FAK and PYK2 signaling along with chemokine or growth factor receptor pathways will prove therapeutically efficacious alone or in the context of conventional chemotherapeutic approaches.

## Methods

### Mice

LN3 (gift from T. Serwold)^32^, CD2-*Lmo2* transgenic (LMO2; gift from U. Davé)^43^, B6.129S4-*Icam1*^*tm1Jcgr*^/J (*Icam1*^-/-^)^62^, C57BL/6 J, and B6.SJL-*Ptprc*^*a*^ *Pepc*^*b*^/BoyJ (CD45.1) mouse strains were bred in-house and housed in specific-pathogen-free conditions. Mice were housed in a 12-h dark/light cycle, in ventilated cages, under regularly monitored temperature (68–76 °F) by veterinary staff. Mice were sourced from The Jackson Laboratory, unless otherwise noted. All experimental procedures were approved under protocol AUP-2019-00034 and AUP-2022-00045 by the Institutional Animal Care and Use Committee (IACUC) at the University of Texas at Austin.

### T-ALL transplant model

For all cellular assays, a total of 5 × 10^6^ primary T-ALL cells, isolated from spleens of leukemic CD45.2^+^ LN3 or LMO2 mice, were resuspended in sterile phosphate-buffered saline (PBS), and injected intraperitoneally (i.p.) into nonirradiated 6–8-week-old sex-matched CD45.1 *Icam1*^-/-^, CD45.1, or CD45.1/CD45.2 mice, as indicated. Both sexes of recipient mice were used as we have not observed sex-based differences in T-ALL engraftment, progression, or myeloid dependence. T-ALL engraftment (CD45.2^+^CD5^+^) in the spleens or tail blood was evaluated by flow cytometry to confirm tumor establishment. The health of the mice was monitored on a daily basis, and changes in behavior indicating distress, including labored breathing, reduced ambulation, feeding, drinking and self-grooming, were taken as humane endpoints for euthanasia in accordance with our IACUC-approved protocol at the University of Texas at Austin.

### Flow cytometric analysis

Organs were harvested and processed as follows: Spleens and thymi were mechanically dissociated with FACS wash buffer (FWB: phosphate-buffered saline (PBS) supplemented with 2% (v/v) BCS (Bovine Calf Serum; GemCell) and 0.5 mM EDTA). Bone marrow cells were obtained by flushing femurs with 2 ml of FWB. Inguinal lymph nodes were enzymatically digested with a cocktail of 0.6 mg/ml (w/v) Liberase and 20 U/ml DNase I (both from Roche). LNs were sequentially digested 3 times with 2 ml of cocktail for 12 min per digest. Livers were mechanically dissociated, spun at 50 g for 3 min, and the supernatant was collected and analyzed. All single-cell suspensions were filtered using 40 µm filters (Fisher Scientific) and subjected to red blood cell lysis using RBC Lysis Buffer (BioLegend). Cells were immunostained by incubating at 4°C for 30 min with fluorescently labeled antibodies below (all antibodies were purchased from BioLegend, unless otherwise indicated). After staining, cells were washed 1-2 times in FWB and resuspended in FWB containing 1 µg/ml propidium iodide (PI; Enzo Life Sciences) to assess viability. All flow cytometric data were acquired using an LSR II flow cytometer (BD Biosciences). Post-acquisition data analysis was performed using FlowJo (v9.9.6 and v10; Tree Star, Inc.).

For mouse lymphoid staining, anti-mouse CD4-PerCP/Cy5.5 (RM4-5; 1:200), CD5-PE (53-7.3; 1:200), CD8-Pacific Blue (53-6.7; 1:200), CD45.1-FITC (A20; 1:100), CD45.2-APC/Cy7 (104; 1:100), B220-Alexa Fluor 700 (RA3-6B2; 1:200), and TCRβ-PE/Cy7 (H57-597; 1:200) antibodies were used. For mouse myeloid staining, CD64-PE (X54-5/7.1; 1:200), CD11c-Pacific Blue (N418; 1:200), I-A/I-E-APC/Cy7 (M5/114.15.2; 1:200), CD172a-PE/Cy7 (SIRPα; P84; 1:200), biotinylated XCR1 (ZET; 1:200), CD11b-Alexa Fluor 700 (M1/70; 1:200), CD115-Alexa Fluor 488 (AFS98; 1:200), Gr1-Brilliant Violet 510 (RB6-8C5; 1:200), F4/80-Alexa Fluor 647 (T45-2342; BD Biosciences; 1:200), and Qdot 605 streptavidin conjugate (Invitrogen; 1:200) antibodies were used. For mouse integrin staining, anti-mouse CD11a-FITC (ITGαL; 2D7; 1:200), CD18-PE (ITGβ2; M18/2; 1:200), CD29-PE (ITGβ1; HMβ1-1; 1:200), CD49d-Alexa Fluor 488 (ITGα4; R1-2; 1:200), Integrin α9 (N-19; Santa Cruz Biotechnology; 1:20), and donkey anti-goat IgG-Alexa Fluor 488 (Jackson ImmunoResearch; 1:400) antibodies were used. For mouse adhesion molecule staining, anti-mouse CD54-PerCP/Cy5.5 (ICAM-1; YN1/1.7.4; 1:200) and CD106-PerCP/Cy5.5 (VCAM-1; 429(MVCAM.A); 1:200) antibodies were used. The relevant isotype antibodies (RTK2758, RTK4530, HTK888, and Poly24030) were used as controls.

For human myeloid staining, anti-human CD11b-Alexa Fluor 700 (M1/70; 1:200) and CD14-PerCP/Cyanine5.5 (M5E2; 1:200) were used. For human integrin staining, anti-human CD11a-PE (HI111; 1:200), CD18-FITC (TS1/18; 1:200), CD29-Alexa Fluor 488 (TS2/16; 1:200), CD49d-PE (9F10; 1:200), and Integrin α9β1-PE (Y9A2; 1:200) antibodies were used. For human adhesion molecule staining, anti-human CD54-FITC (HA58; 1:200), CD106-APC or -PE (STA; 1:200), CD106 (AF809; R&D Systems; 1:200), and donkey anti-sheep IgG-Alexa Fluor 488 (Invitrogen; 1:400) antibodies were used. The relevant isotype antibodies (MOPC-21 and 31243 (Invitrogen)) were used as controls.

For intracellular immunostaining of proteins, single-cell suspensions were labeled with LIVE/DEAD Fixable Dead Cell Stain Kit (Green or Aqua; Invitrogen) and then treated with FIX & PERM Fixation & Cell Permeabilization Kit (Invitrogen) according to the manufacturer’s methanol modification procedure. Cells were then stained with fluorescently labeled antibodies against anti-mouse CD45.1-PerCP/Cy5.5 (A20; 1:100), CD45.2-APC/Cy7 (104; 1:100), CD5-PE/Cy7 (53-7.3; 1:200), pIGF1R-Alexa Fluor 647 (K74-218; pY1131; BD Biosciences; 1:10), pAKT-PE or -Brilliant Violet 421 (J24-618; pS473; BD Biosciences; 1:10), pPYK2-PE (L68-1256.272; pY402; BD Biosciences; 1:10), pFAK (#3283; pY397; Cell Signaling Technology; 1:200), pFAK (#3284; pY925; Cell Signaling Technology; 1:50), pFAK (44–626 G; pY861; Invitrogen; 1:200), donkey anti-rabbit IgG-PE (Poly4064; 1:400), ILK (P83A9; 1:200), donkey anti-rabbit IgG-Alexa Fluor 488 (Jackson ImmunoResearch; 1:400) and goat anti-mouse IgG-Alexa Fluor 488 (Invitrogen; 1:400) antibodies. The relevant isotype antibodies (MOPC-21, MPC-11, and Poly29108) were used as negative controls.

### Transwell assays

Enriched T-ALL cells and/or myeloid cells from the spleens of mice engrafted with primary LN3 T-ALL were plated in the top or bottom chambers of 12 mm Transwell plates with 0.4 µm pore polyester membrane cell culture inserts (Corning) as indicated in Fig. 1a. 1 × 10^6^ T-ALL cells or 5 × 10^4^ myeloid cells were plated in the top chamber of the inserts, while 3 × 10^6^ T-ALL cells +/- 1.5  × ;10^5^ myeloid cells were plated in bottom chamber, with 500 µl or 1.5 ml of complete RMPI, respectively. The viability of T-ALL cells in each chamber was assessed by flow cytometry after 6–7 days of culture.

### In vitro cultures of mouse and human T-ALL cells

Complete RPMI cell culture media was made up of Roswell Park Memorial Institute 1640 (RPMI 1640) supplemented with 10% fetal bovine serum (FBS; Gemini Bio-Products), 55 µM β-mercaptoethanol, 1 × GlutaMAX (2 mM L-alanyl-L-glutamine dipeptide), 1 mM Sodium Pyruvate, 1 × MEM Non-essential Amino Acid Solution (Sigma-Aldrich), 1 × Penicillin-Streptomycin-Glutamine (100 units of penicillin, 100 µg of streptomycin, and 292 µg/ml of L-glutamine). All reagents were obtained from Gibco, unless otherwise indicated.

For enrichment of mouse T-ALL cells, single-cell suspensions of leukemic spleens from mice engrafted with primary T-ALL were incubated with biotinylated antibodies against anti-mouse CD11b (M1/70), CD11c (N418), F4/80 (BM8), and I-A/I-E (M5/1114.15.2), and negatively enriched with MojoSort Streptavidin Nanobeads, according to the manufacturer’s instructions. For preparation of tumor-associated myeloid cells, single-cell suspensions of leukemic spleens were incubated with biotinylated antibodies against CD11c and positively isolated using Streptavidin Microbeads and MACS LS Columns (both from Miltenyi Biotec). 3 × 10^5^ T-ALL cells were cultured in the presence or absence of 2–2.5 × 10^4^ enriched myeloid cells in 200 µl of complete RMPI in flat-bottom 96-well plates at 37˚C, 5% CO_2_ for 6–7 days. All reagents were obtained from BioLegend, unless otherwise indicated.

For culture of human T-ALL cells, 8 de-identified primary pediatric T-ALL samples were obtained from Texas Children’s Hospital (Houston, TX). Informed consent from the parent and assent from children over the age of 8 years were obtained, and compensation was not provided. T-ALL specimens were provided by children who ranged in age from 2–13 years, were black or caucasian, hispanic or non-hispanic, and all but one were male. Sample procurement and analysis were approved by the institutional review board committees at The University of Texas at Austin and Texas Children’s Hospital/Baylor College of Medicine. Patient T-ALL cells were isolated by density gradient separation using Ficoll, washed, and frozen in RPMI 1640 supplemented with 40% (v/v) FBS and 10% dimethyl sulfoxide (DMSO) upon collection. Human myeloid cells were prepared as previously reported.^16^ Briefly, leukoreduction system chambers were obtained from We Are Blood (Austin, TX) and peripheral blood mononuclear cells (PBMCs) were isolated by density gradient separation using Histopaque (1.077 g/ml; Sigma-Aldrich). For enrichment of monocytes, 40 × 10^6^ PBMCs were plated in 150 × 15 mm Petri Dish (Corning) in 25 ml of complete RPMI and incubated at 37˚C, 5% CO_2_ for 2 h, after which adherent monocytes were collected using cold PBS and cell scrapers (Fisher Scientific). For enhanced purity, collected monocytes were incubated with biotinylated antibodies against human CD3 (UCHT1; BioLegend) and CD19 (HIB19; BioLegend) and lymphocytes were depleted with MojoSort Streptavidin Nanobeads (BioLegend), according to the manufacturer’s instructions. For differentiation of macrophages, 40 × 10^6^ PBMCs were seeded into T75 flasks (Corning) in 20 mL of complete RPMI medium and cultured at 37 °C, 5% CO_2_ for 2 h to allow for monocyte adhesion. After adherent monocytes were washed twice with warm complete RPMI medium, monocytes were differentiated for 7–9 days in the presence of macrophage colony-stimulating factor (M-CSF; 50 ng/mL; PeproTech). M-CSF-derived macrophages were collected using cold PBS and cell scrapers (Fisher Scientific). For co-cultures, 1 × 10^5^ patient T-ALL cells were plated alone, with monocytes, or with M-CSF-derived macrophages at a 1:1 or 2:1 ratio, respectively, in 200 μL of complete RPMI in flat-bottom 96-well plates for 6–7 days prior to assessing the viability of T-ALL cells by flow cytometry.

To determine whether blockade of integrin-mediated cell adhesion inhibits myeloid-mediated support of mouse T-ALL, 3 × 10^5^ T-ALL cells isolated from primary T-ALL-engrafted mice were co-cultured with 2–2.5 × 10^4^ enriched tumor-associated myeloid cells in the presence or absence of blocking antibodies against integrins (anti-mouse CD11a (M17/4) and/or CD29 (HMβ1-1)) or adhesion molecules (anti-mouse CD54 (YN1/1.7.4) and/or CD106 (429(MVCAM.A))). The relevant isotype antibodies (RTK2758, RTK4530, and HTK888, 53-6.7 (anti-mouse CD8), and 7E.17G9 (anti-mouse ICOS)) were used as controls.

To determine whether inhibition of integrin-mediated cell adhesion suppresses myeloid-mediated support of human T-ALL, 1 × 10^5^ T-ALL cells were plated with monocytes or M-CSF-derived macrophages at a 1:1 or 2:1 ratio, respectively, in the presence or absence of a cocktail of anti-human CD54 (HCD54; 20 µg/ml) and CD106 (AF809; R&D Systems) or anti-human CD11a (HI111) and CD29. The relevant isotype antibodies (MOPC-21, 31243 (Invitrogen), and OKT-6 (anti-human CD1a; BioXCell; 30 µg/ml)) were used as controls. The final concentration of each antibody is 10 µg/ml, unless otherwise indicated, and all the antibodies above were purchased from BioLegend, unless otherwise indicated.

To determine whether blockade of integrin-mediated close contact inhibits sensitization of mouse T-ALL cells to exogenous IGF1 by tumor-associated myeloid cells, 3 × 10^5^ T-ALL cells were cultured in the presence or absence of 2–2.5 × 10^4^ enriched tumor-associated myeloid cells, with or without blocking antibodies against mouse adhesion molecules (above), and in the presence or absence of 100 ng/ml of recombinant murine IGF1 (Peprotech).

To determine whether inhibition of integrin-mediated close contact diminishes IGF1R and AKT signaling, 1 × 10^6^ mouse T-ALL cells were co-cultured with 7–8 × 10^4^ myeloid cells in the presence of an anti-mouse ICAM antibody (YN1/1.7.4) and/or an anti-mouse VCAM antibody (429(MVCAM.A)), or the relevant isotype controls, at 10 µg/ml each in 500 µl of complete RPMI in flat-bottom 48-well plates. On day 3–4 of culture, cells in each group were intracellularly stained for phosphorylated proteins as described above.

To determine whether survival of mouse or human T-ALL cells co-cultured with myeloid cells requires IGF1R and focal adhesion signaling (mouse: 3 × 10^5^ T-ALL cells were cultured in the presence or absence of 2–2.5 × 10^4^ enriched tumor-associated myeloid cells / human: 1 × 10^5^ T-ALL cells were cultured with monocytes or M-CSF-derived macrophages at a 1:1 or 2:1 ratio, respectively), cells were cultured for 4–5days prior to addition of 0.1–2.5 µM FAK/PYK2 dual inhibitors (PF-431396 and PF-562271; Selleckchem). Viability and pIGF1R levels were assessed by flow cytometry 3–4 days after inhibitor addition in comparison to cells treated with vehicle control (DMSO).

### In vivo inhibition of integrin-mediated cell adhesion or FAK/PYK2 signaling

*Icam1*^-/-^ or *Icam1*^+/-^ littermate controls (6–8 weeks old) were engrafted i.p. with 5 × 10^6^ primary LN3 T-ALL cells. At 1-2% blood chimerism in *Icam1*^+/-^ mice, T-ALL burden and IGF1R and AKT activation were assessed in multiple organs by flow cytometry. Survival was monitored in separate cohorts. To determine whether VCAM-1 blockade further deceased T-ALL survival in vivo, *Icam1*^-/-^ or *Icam1*^+/-^ littermate controls with established T-ALL burden ( > 1% splenic chimerism) were randomly divided into two groups, respectively, and treated i.p. with 100 µg of *InVivo*MAb anti-mouse VCAM-1 (M/K-2.7) or respective isotype control (HRPN) antibodies in 200 µl of PBS every 2 days for a total of five injections. T-ALL burden and IGF1R and AKT activation were assessed by flow cytometry 48 h after the final injection.

For antibody-mediated blockade of adhesion molecules, congenic mice with established T-ALL burden ( > 1% splenic chimerism) were randomly divided into three groups and treated i.p. with 100 µg each of *InVivo*MAb antibodies in 200 µl of PBS every 2 days for a total of five injections: (1) anti-mouse ICAM-1 (YN1/1.7.4) alone, (2) a cocktail of anti-mouse CD54 and CD106 (M/K-2.7), or (3) respective isotype controls (HRPN and LTF-2; all InVivoMAb antibodies are from BioXCell). T-ALL burden was evaluated 24–48 h after the final injection.

For inhibition of FAK/PYK2 signaling, congenic mice with established T-ALL burden ( > 0.2% splenic chimerism) were randomly divided into treated and control groups. PF-562271 (Selleckchem) was administered i.p. at 25 mg/kg body weight in 200 ul injection solution once daily for 10–11 consecutive days. Injection solution was comprised of 10% PF-562271 (or DMSO), 40% PEG300 (Selleckchem), 5% TWEEN 80 (Sigma-Aldrich), and 45% PBS. T-ALL burden was evaluated 1–3 h after the final injection. All comparisons within experiments were carried out with age-matched mice (6–8 weeks old) engrafted with the same primary LN3 T-ALL.

### Quantitative PCR

Thymic T-ALL cells were resuspended in TRIzol (Invitrogen), RNA was extracted, and cDNA was generated using qScript cDNA SuperMix (Quantabio). Real-time PCR experiments were performed on an Applied Biosystems Viia7 instrument with the 7500 Fast real-time PCR system using the following primers: beta-actin forward 5'-CACTGTCGAGTCGCGTCCA-3', beta-actin reverse 5'-CATCCATGGCGAACTGGTGG-3', ITGα4 forward 5'- GCA GAG TCT CCG TCA AGA TTT-3', ITGα4 reverse 5'- CCT GGT GTG TCC TAC ATT TCT C-3', ITGα9 forward 5'- TCC CTG CTA CGA AGA GTA TAA GA-3', ITGα9 reverse 5'- GAG TGT CCC AGC CCA ATA AA-3', ITGαL forward 5'- GCC TAT CCT GAG ACC TTC AAT C-3', ITGαL reverse 5'- AGG TTT GCC TCA CAC TTC TT-3', ITGβ1 forward 5'-GAC AGT GTG TGT GTA GGA AGA G-3', ITGβ1 reverse 5'-GCC TCC ACA AAT TAA GCC ATT AG-3', ITGβ2 forward 5'- GTG GTA GGT GTC GTA CTG ATT G-3', ITGβ2 reverse 5'- GGG ACT TGA GTT TCT CCT TCT C-5'.

### Bioinformatic analysis of mouse T-ALL RNA-seq data

Our published RNA-seq results of LN3 T-ALL vs healthy T-lineage cells were re-analyzed. In brief, FastQ files were assessed for quality using FastQC. Reads were then pseudo-aligned or aligned to the mouse transcriptome (GRCm38) to estimate transcript abundances using Kallisto (v0.46.1)^63^ or HISAT2 (v.2.0.4)^64^ respectively. We found they perform similarly, as has been reported^65^. The transcript-level counts were aggregated to gene-level counts using tximport in R^66^, normalized using DESeq2^67^ and size factors were transformed with variance stabilizing transformation to yield counts that are approximately homoscedastic. Genes that correlated significantly (absolute fold change ≥ 2, adjusted *P*-value < 0.05) to sequencing batch were identified using the likelihood ratio test in DESeq2 and were removed from further analysis. To determine processes upregulated in T-ALL, we compared thymic T-ALL and healthy thymocytes using DESeq2, yielding a list of upregulated genes (fold change ≥ 2, adjusted *P*-value < 0.05), and carried out functional enrichment analyses using Metascape^68^ with Gene Ontology (GO) Biological Processes, Kyoto Encyclopedia of Genes and Genomes (KEGG) Pathways, Reactome gene sets, and WikiPathways; default cutoffs were used for enrichment (minimum overlap = 3, *P*-value cutoff = 0.01, minimum enrichment = 1.5). To perform pairwise comparisons of T-ALL cells vs healthy T-lineage cells in the spleens or thymuses separately or comparisons between healthy T-lineage cells in the spleen vs thymus, Gene Set Enrichment Analysis (GSEA) was performed on differentially expressed genes identified by DESeq2 (absolute fold change ≥ 1.5, adjusted *P*-value < 0.05) using the PID gene sets with gene set permutations (1000) and default parameters (enrichment statistic: weighted, metric for ranking: signal2noise, gene list sorting model: real, gene list ordering mode: descending, maximum size:500, minimum size:15). The top 15 pathways significantly enriched or depleted in T-ALL cells relative to controls in each organ were visualized using the pheatmap package in R (The R Foundation for Statistical Computing). Further pathway analyses using the WikiPathways 2019 Mouse gene sets of Enrichr was run on all genes highly expressed in T-ALL cells in the thymuses relative to thymocytes (fold change ≥ 1.5, adjusted *P*-value < 0.05)^69–71^.

### Bioinformatic analysis of patient T-ALL RNA-seq data

Bioinformatic analysis was performed on published RNA-seq data of T-ALL samples from 264 patients and the associated clinical outcomes datasets from the TARGET ALL Phase II project. Patients whose WBC numbers at diagnosis were >200,000/ml were excluded. We used xCell^72^ to quantify the enrichment scores of macrophages and monocytes from bulk RNA-seq data. Pathway information related to integrin signaling was obtained from MSigDB using the R package “msigdbr^73^.” The enrichment scores of the integrin pathways in each RNA-seq sample were calculated using the “fgsea” function from the “fgsea” R package^74^. For calculation of FAK signaling signature scores, we downloaded the transcriptomics data of FAK-WT and FAK-null murine squamous cell carcinoma cells from Gene Expression Omnibus (GEO; GSE147670), and performed differential expression analyses, identifying the top 50 most upregulated genes in FAK-WT cells. The 50 mouse genes were mapped to human orthologs using the biomaRt package^75^. Using the 50-gene list as an expression signature of FAK signaling, we calculated signature scores for each patient sample. To plot a longitudinal event-free survival of pediatric T-ALL patients, we divided patient data into high and low groups for each integrin or FAK pathway using the median as a cutoff value. Log-rank tests were used to detect the correlation of the integrin or FAK pathways with the event free survival time of T-ALL patients. Pearson correlations were calculated to assess the correlation between each myeloid subset (monocytes or M-CSF-derived macrophages) and integrin or FAK pathways. R was used for the data analyses.^76^

### Statistical analysis

Statistical analyses were performed with Prism (v9; GraphPad Software). Normality was determined using the D’Agostino & Pearson or Shapiro–Wilk tests, as appropriate for sample size. Statistical significance was determined using unpaired Student *t*-tests for normally distributed data or the non-parametric Mann-Whitney test, paired Student *t*-tests, repeated measures one-way ANOVA with the Holm-Sidak correction for normally distributed data or the non-parametric Friedman test, two-way ANOVA with the Holm-Sidak correction, or Log-rank tests, as indicated in figure legends.

### Reporting summary

Further information on research design is available in the Nature Portfolio Reporting Summary linked to this article.

### Supplementary information

Supplementary Information

Reporting Summary

## Data Availability

The data generated in this study are available within the article and its supplementary data files, and source data are provided with this paper. The mouse RNA-seq data analyzed in this study were obtained from Gene Expression Omnibus (GEO) at GSE150096. The patient RNA-seq data analyzed in this study were obtained from the TARGET website at https://ocg.cancer.gov/programs/target. The transcriptomics data of FAK-WT and FAK-null murine squamous cell carcinoma cells were obtained from GEO at GSE147670.
