# Supplementary material for: Integrin signaling is critical for myeloid-mediated support of T-cell acute lymphoblastic leukemia
Source: Nat Commun. 2023 Oct 7;14:6270. doi: 10.1038/s41467-023-41925-z (PMC10560206; doi:10.1038/s41467-023-41925-z)
Supplement: Supplementary file 1 — Supplementary Information [file 41467_2023_41925_MOESM1_ESM.pdf]

Supplementary Information

Integrin signaling is critical for myeloid-mediated support of T-cell acute lymphoblastic leukemia

Supplementary Figures

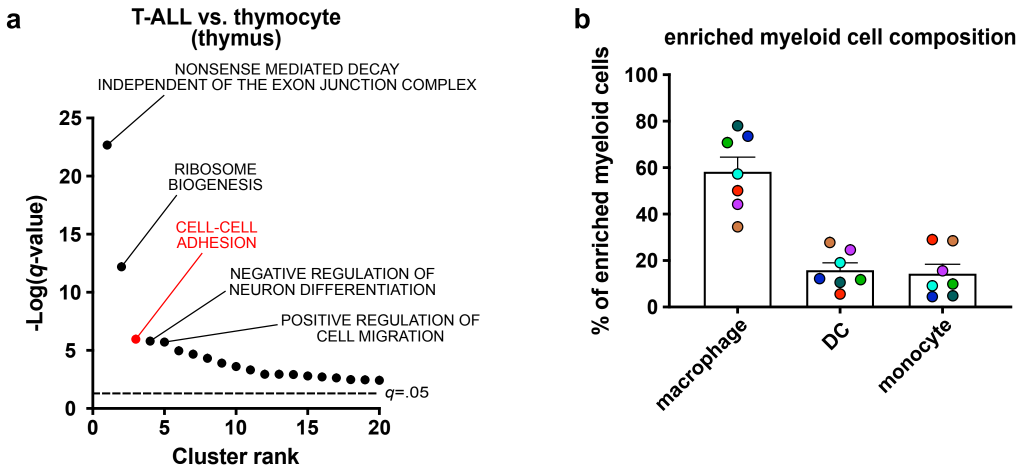

Supplementary Figure 1. Close contact is critical for myeloid-mediated T-ALL support.

(a) Plot of term clusters significantly enriched among differentially expressed genes from thymic T-ALL cells relative to healthy thymocytes, as identified using the Metascape bioinformatics tool. The "cell-cell adhesion" cluster is indicated in red among the top 5 clusters. (b) Quantification of the frequency of the indicated subsets within enriched myeloid cells used for co-cultures from leukemic spleens. Bars represent mean + SEM from n=7 independent experiments with distinct color-coded tumors; symbols represent individual mice from each experiment.

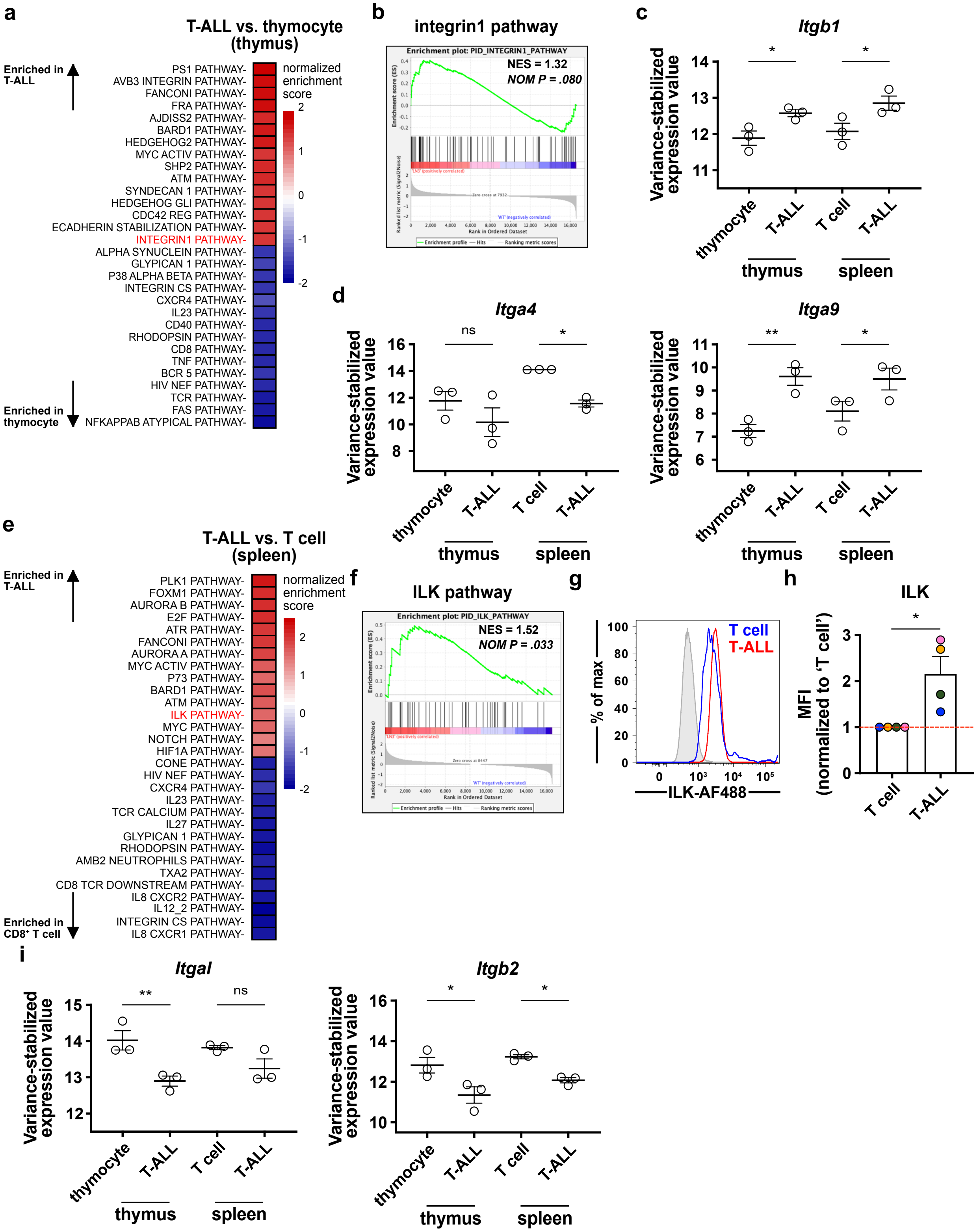

a
T-ALL vs. thymocyte (thymus)
Enriched in T-ALL
Enriched in thymocyte
PS1 PATHWAY-
AVB3 INTEGRIN PATHWAY-
FANCONI PATHWAY-
FRA PATHWAY-
AJDISS2 PATHWAY-
BARD1 PATHWAY-
HEDGEHOG2 PATHWAY-
MYC ACTIV PATHWAY-
SHP2 PATHWAY-
ATM PATHWAY-
SYNDECAN 1 PATHWAY-
HEDGEHOG GLI PATHWAY-
CDC42 REG PATHWAY-
ECADHERIN STABILIZATION PATHWAY-
INTEGRIN1 PATHWAY-
ALPHA SYNUCLEIN PATHWAY-
GLYPICAN 1 PATHWAY-
P38 ALPHA BETA PATHWAY-
INTEGRIN CS PATHWAY-
CXCR4 PATHWAY-
IL23 PATHWAY-
CD40 PATHWAY-
RHODOPSIN PATHWAY-
CD8 PATHWAY-
TNF PATHWAY-
BCR 5 PATHWAY-
HIV NEF PATHWAY-
TCR PATHWAY-
FAS PATHWAY-
NFKAPPAB ATYPICAL PATHWAY-
normalized enrichment score
2
1
0
-1
-2
b
integrin1 pathway
Enrichment plot: PID_INTEGRIN1_PATHWAY
NES = 1.32
NOM P = .080
c
Itgb1
Variance-stabilized expression value
14
13
12
11
*
*
thymocyte
T-ALL
T cell
T-ALL
thymus
spleen
d
Itga4
Variance-stabilized expression value
16
14
12
10
8
ns
*
thymocyte
T-ALL
T cell
T-ALL
thymus
spleen
Itga9
Variance-stabilized expression value
11
10
9
8
7
6
**
*
thymocyte
T-ALL
T cell
T-ALL
thymus
spleen
e
T-ALL vs. T cell (spleen)
Enriched in T-ALL
Enriched in CD8⁺ T cell
PLK1 PATHWAY-
FOXM1 PATHWAY-
AURORA B PATHWAY-
E2F PATHWAY-
ATR PATHWAY-
FANCONI PATHWAY-
AURORA A PATHWAY-
MYC ACTIV PATHWAY-
P73 PATHWAY-
BARD1 PATHWAY-
ATM PATHWAY-
ILK PATHWAY-
MYC PATHWAY-
NOTCH PATHWAY-
HIF1A PATHWAY-
CONE PATHWAY-
HIV NEF PATHWAY-
CXCR4 PATHWAY-
IL23 PATHWAY-
TCR CALCIUM PATHWAY-
IL27 PATHWAY-
GLYPICAN 1 PATHWAY-
RHODOPSIN PATHWAY-
AMB2 NEUTROPHILS PATHWAY-
TXA2 PATHWAY-
CD8 TCR DOWNSTREAM PATHWAY-
IL8 CXCR2 PATHWAY-
IL12_2 PATHWAY-
INTEGRIN CS PATHWAY-
IL8 CXCR1 PATHWAY-
normalized enrichment score
2
1
0
-1
-2
f
ILK pathway
Enrichment plot: PID_ILK_PATHWAY
NES = 1.52
NOM P = .033
g
% of max
100
80
60
40
20
0
T cell
T-ALL
0
10³
10⁴
10⁵
ILK-AF488
h
ILK
MFI (normalized to 'T cell')
3
2
1
0
*
T cell
T-ALL
i
Itgal
Variance-stabilized expression value
15
14
13
12
**
ns
thymocyte
T-ALL
T cell
T-ALL
thymus
spleen
Itgb2
Variance-stabilized expression value
16
14
12
10
*
*
thymocyte
T-ALL
T cell
T-ALL
thymus
spleen

Supplementary Figure 2. Integrin-associated pathways are enriched in T-ALL cells relative to tumor-free T-lineage cells.

(a) The top 15 pathways significantly enriched or depleted in thymic T-ALL cells relative to tumor-free thymocytes were identified by the Gene Set Enrichment Analysis (GSEA) bioinformatics tool using PID gene sets. (b) GSEA enrichment plots for the INTEGRIN1 pathway in thymic T-ALL cells relative to tumor-free thymocytes as in (a). Normalized enrichment scores (NES) and nominal *P*-values are shown. (c-d) Variance-stabilized expression values of (c) *Itgb1* and (d) *Itga4* and *Itga9* in tumor-free T-lineage and T-ALL cells from the thymus and spleen, as indicated. Bars represent mean +/- SEM; symbols represent individual biologic replicates. (e) The top 15 pathways significantly enriched or depleted in splenic T-ALL cells relative to tumor-free splenic $CD8^+$ T cells were identified by the GSEA bioinformatics tool using the PID gene sets. (f) GSEA enrichment plots for the ILK pathway in splenic T-ALL cells relative to tumor-free $CD8^+$ T cells as in (e). (g) Representative flow cytometry plots of ILK expression in transplanted LN3 T-ALL cells (red) and host T cells (blue) from the same leukemic spleens. Isotype control stain is shaded in gray. (h) Quantification of the levels of ILK in T-ALL cells ($CD45.2^+CD5^+$) relative to host T cells ($CD45.1^+CD5^+$) from data as in (h), displayed as mean fluorescence intensities (MFI). Results were normalized to the MFI of T cells within each experiment. Bars represent the mean + SEM from n=4 independent experiments, each with a distinct color-coded primary T-ALL. The red line indicates the normalized mean MFI of T cells. (i) Variance-stabilized expression values of *Itgal* and *Itgb2* in tumor-free T-lineage and T-ALL cells from the thymus and spleen, as indicated. Bars represent mean +/- SEM; symbols represent individual biologic replicates. Statistical significance was determined by (c, d, i) a two-way ANOVA with the Holm-Sidak correction, and (h) two-way paired Student t tests; *P*-values: *<0.05, **<0.01. ns, not significant.

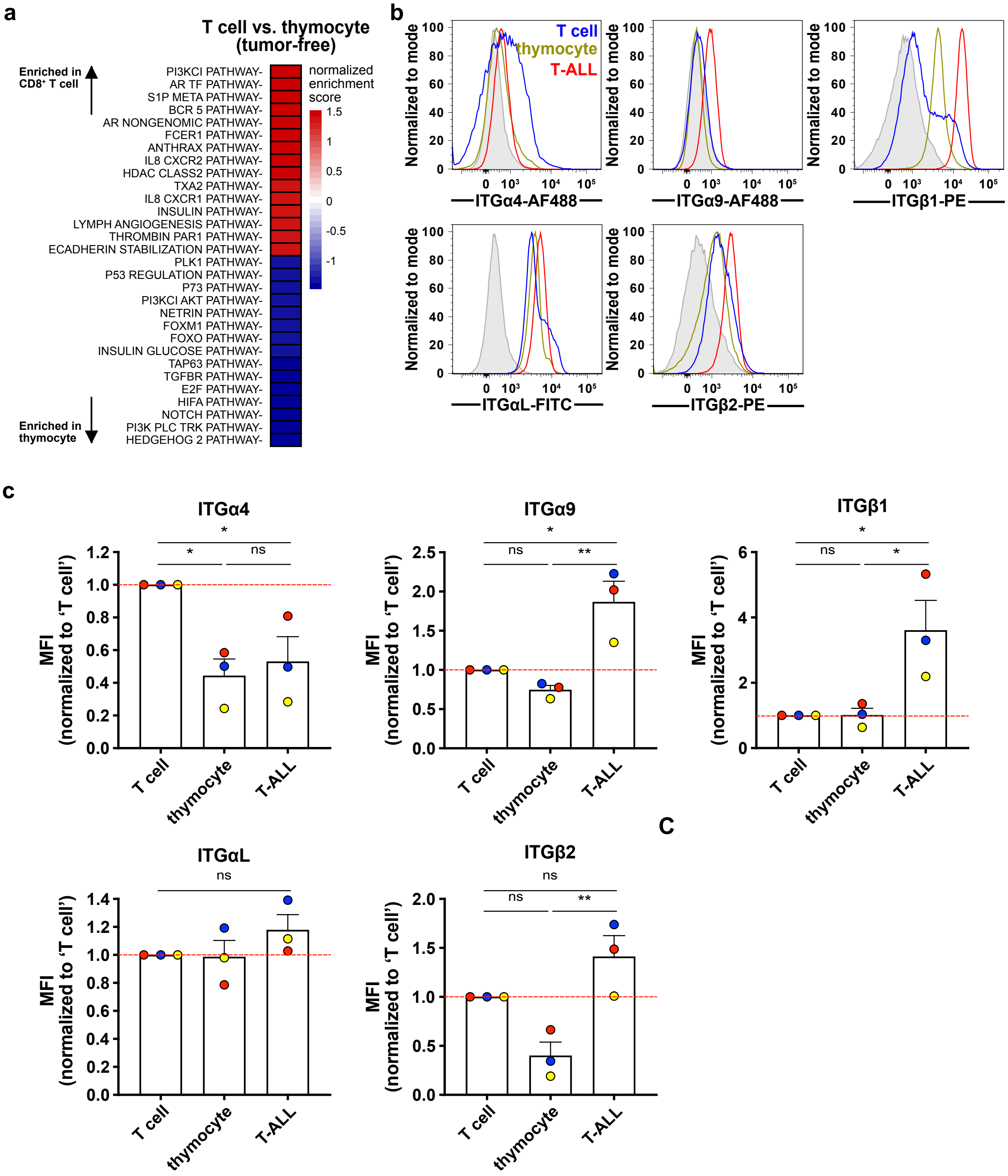
a
T cell vs. thymocyte (tumor-free)
Enriched in CD8⁺ T cell
Enriched in thymocyte
normalized enrichment score
PI3KCI PATHWAY
AR TF PATHWAY
S1P META PATHWAY
BCR 5 PATHWAY
AR NONGENOMIC PATHWAY
FCER1 PATHWAY
ANTHRAX PATHWAY
IL8 CXCR2 PATHWAY
HDAC CLASS2 PATHWAY
TXA2 PATHWAY
IL8 CXCR1 PATHWAY
INSULIN PATHWAY
LYMPH ANGIOGENESIS PATHWAY
THROMBIN PAR1 PATHWAY
ECADHERIN STABILIZATION PATHWAY
PLK1 PATHWAY
P53 REGULATION PATHWAY
P73 PATHWAY
PI3KCI AKT PATHWAY
NETRIN PATHWAY
FOXM1 PATHWAY
FOXO PATHWAY
INSULIN GLUCOSE PATHWAY
TAP63 PATHWAY
TGFBR PATHWAY
E2F PATHWAY
HIFA PATHWAY
NOTCH PATHWAY
PI3K PLC TRK PATHWAY
HEDGEHOG 2 PATHWAY
b
T cell
thymocyte
T-ALL
Normalized to mode
ITGα4-AF488
ITGα9-AF488
ITGβ1-PE
ITGαL-FITC
ITGβ2-PE
c
ITGα4
ITGα9
ITGβ1
ITGαL
ITGβ2
MFI (normalized to 'T cell')
T cell
thymocyte
T-ALL
ns
C

Supplementary Figure 3. T-ALL cells express elevated integrin protein levels relative to healthy T-cell lineage controls in the spleen and thymus.
(a) The top 15 pathways significantly enriched or depleted in healthy splenic $CD8^+$ T cells relative to healthy thymocytes were identified by the Gene Set Enrichment Analysis (GSEA) bioinformatics tool using the PID gene sets. (b-c) (b) Representative flow cytometry plots and (c) quantification of cell surface integrin expression levels of transplanted LN3 T-ALL cells from leukemic spleens (red), healthy splenic T cells (blue), and healthy thymocytes (green). Isotype control stains are shaded in gray. (c) Results were normalized to splenic T-cell levels within each experiment. Data are compiled from n=3 independent experiments, each with a distinct color-coded primary T-ALL. Symbols represent individual mice. Statistical significance was determined by (c) a two-way repeated measures one-way ANOVA with the Holm-Sidak correction; *P*-values: *<0.05, **<0.01. ns, not significant.

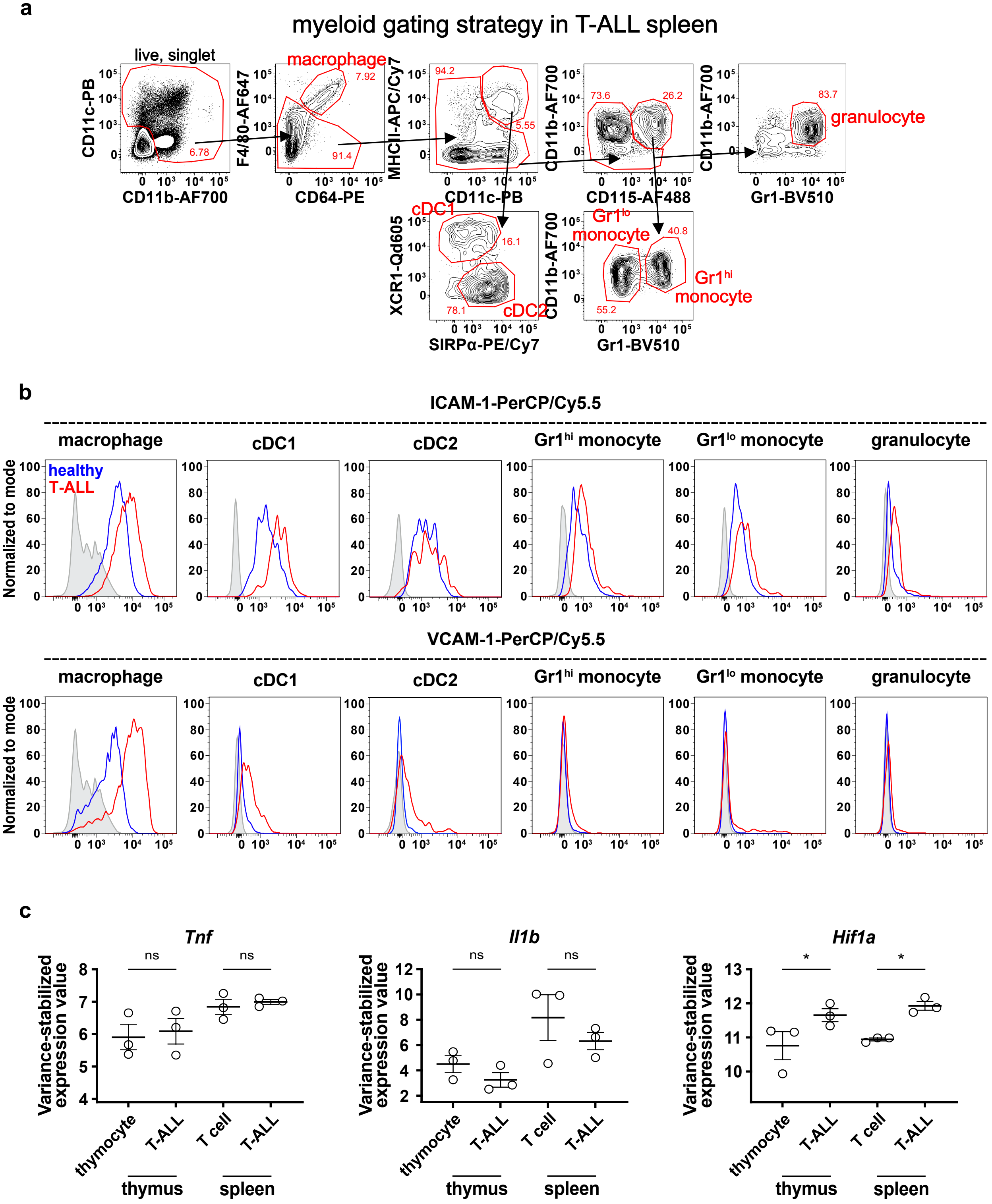
a
myeloid gating strategy in T-ALL spleen
live, singlet
CD11c-PB
CD11b-AF700
6.78
F4/80-AF647
macrophage
7.92
91.4
CD64-PE
MHCII-APC/Cy7
94.2
5.55
CD11c-PB
CD11b-AF700
73.6
26.2
CD115-AF488
83.7
granulocyte
Gr1-BV510
cDC1
XCR1-Qd605
16.1
78.1
cDC2
SIRPα-PE/Cy7
Gr1lo monocyte
40.8
Gr1hi monocyte
55.2
Gr1-BV510
b
ICAM-1-PerCP/Cy5.5
macrophage
cDC1
cDC2
Gr1hi monocyte
Gr1lo monocyte
granulocyte
healthy
T-ALL
Normalized to mode
VCAM-1-PerCP/Cy5.5
c
Tnf
Il1b
Hif1a
Variance-stabilized expression value
ns
*
thymocyte
T-ALL
T cell
thymus
spleen

Supplementary Figure 4. ICAM-1 and VCAM-1 protein levels are elevated in tumor-associated myeloid cells relative to healthy myeloid cells.
(a) Representative sequential gating schemes for evaluation of the indicated myeloid subsets in mice transplanted with primary LN3 T-ALL in the spleen. The initial plot in each gating scheme is pre-gated on live (PI-), singlet cells. Sequential gating is indicated by arrows. (b) Representative flow cytometry histograms of ICAM-1 (top) and VCAM-1 (bottom) expression by the indicated myeloid subsets from the spleens of healthy (blue) or leukemic (red) mice transplanted with primary LN3 T-ALL cells. Isotype control stains are shaded in gray. (c) Variance-stabilized expression values of *Tnf*, *Il1b* and *Hif1a* in tumor-free T-lineage and T-ALL cells from the thymus and spleen, as indicated. Bars represent mean +/- SEM; symbols represent individual biologic replicates. Statistical significance was determined by (c) a two-way ANOVA with the Holm-Sidak correction; *P*-values: *<0.05. ns, not significant.

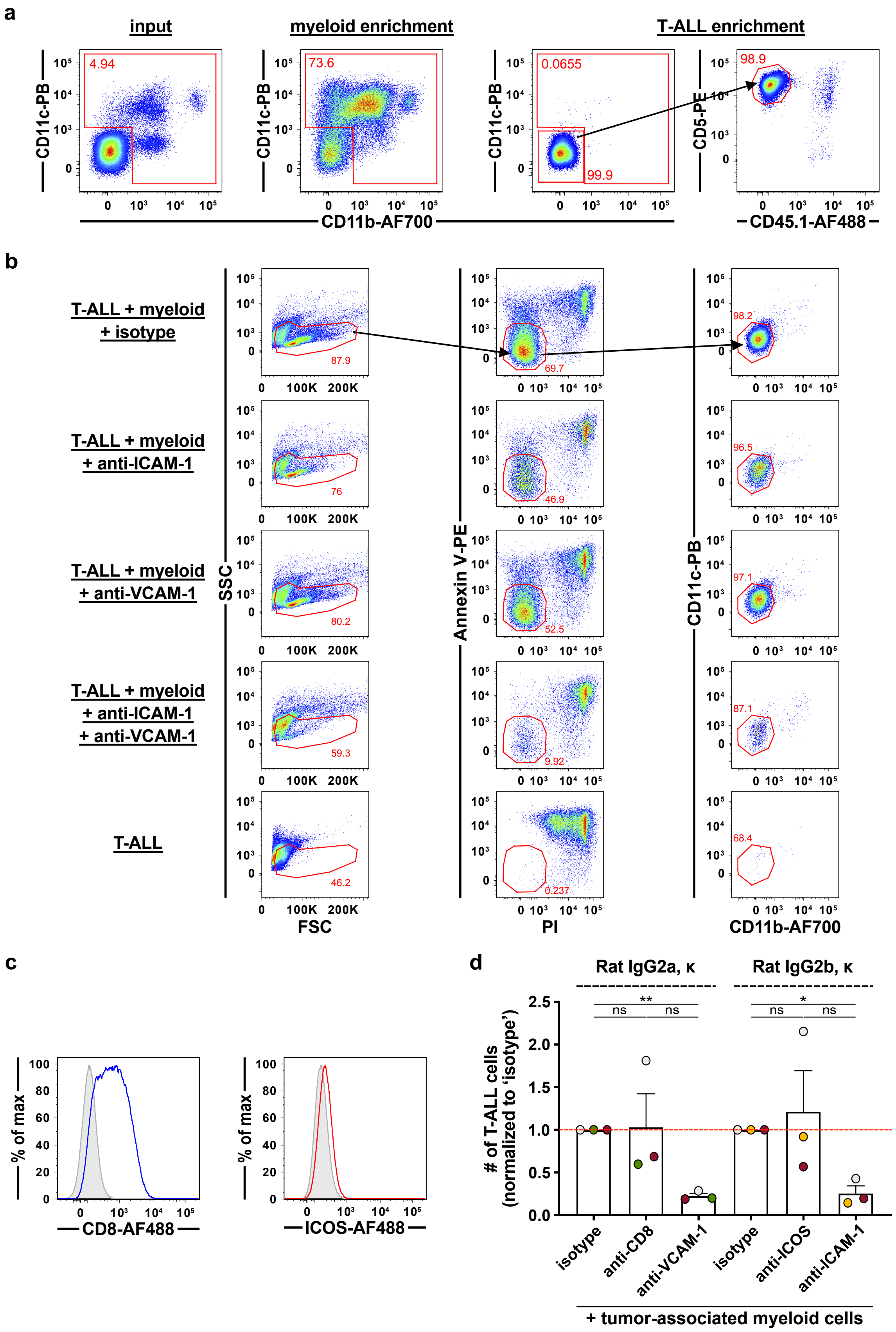

a
input
myeloid enrichment
T-ALL enrichment
CD11c-PB
CD11b-AF700
CD5-PE
CD45.1-AF488
4.94
73.6
0.0655
99.9
98.9
b
T-ALL + myeloid + isotype
T-ALL + myeloid + anti-ICAM-1
T-ALL + myeloid + anti-VCAM-1
T-ALL + myeloid + anti-ICAM-1 + anti-VCAM-1
T-ALL
SSC
FSC
Annexin V-PE
PI
CD11c-PB
CD11b-AF700
87.9
76
80.2
59.3
46.2
69.7
46.9
52.5
9.92
0.237
98.2
96.5
97.1
87.1
68.4
c
% of max
CD8-AF488
ICOS-AF488
d
Rat IgG2a, κ
Rat IgG2b, κ
# of T-ALL cells (normalized to 'isotype')
isotype
anti-CD8
anti-VCAM-1
isotype
anti-ICOS
anti-ICAM-1
ns
**
*
+ tumor-associated myeloid cells

Supplementary Figure 5. Inhibition of ICAM-1- and VCAM-1-mediated cell adhesion reduces T-ALL survival in vitro.

(a) Representative flow cytometry plots showing the purity of enriched tumor-associated myeloid ($CD11b^+$ and/or $CD11c^+$) and T-ALL ($CD11b^-CD11c^-CD5^+CD45.2^+$) cells from the spleens of mice transplanted with primary LN3 T-ALL cells with sequential gating indicated by arrows. “Input” shows myeloid composition ($CD11b^+$ and/or $CD11c^+$) before enrichment. “Myeloid enrichment” shows the percent of myeloid cells following positive enrichment of splenic $CD11c^+$ cells. “T-ALL enrichment” shows the low frequency of myeloid ($CD11b^-$ and $CD11c^-$) cells within the enriched T-ALL after depletion with antibodies against F4/80, CD11b, I-A/I-E, and CD11c (left) and expression of CD5 and CD45.1 on the enriched T-ALL fraction (right). (b) Representative flow cytometry plots showing viability of transplanted LN3 T-ALL cells cultured in the presence or absence of enriched tumor-associated myeloid cells from the spleen, as in (a), and in the presence or absence of anti-ICAM-1 and/or anti-VCAM-1 antibodies. Viable T-ALL cells were quantified as Annexin V $(AV)^-PI^-CD11b^-CD11c^-$ cells. Sequential gating is indicated by arrows. (c) Representative flow cytometry histograms of CD8 (blue) and ICOS (red) expression by T-ALL cells from the spleens of mice transplanted with primary LN3 T-ALL cells. Isotype control stains are shaded in gray. (d) Quantification of viable LN3 T-ALL cells 6-7 days after co-culture with enriched tumor-associated myeloid cells in the presence of anti-CD8 (Rat IgG2a, κ) or anti-ICOS (Rat IgG2b, κ) antibodies (10 μg/ml each). Data from anti-VCAM-1 (Rat IgG2a, κ)- and anti-ICAM-1 (Rat IgG2b, κ)-treated cultures from Figure 2a were included for comparison. Results were normalized to isotype-treated cultures within each experiment. Bars show the mean + SEM from n=3 independent experiments, each with a distinct color-coded primary T-ALL; symbols represent the average of 2-3 technical replicate wells per experiment. The red line indicates the normalized mean viability of isotype-treated T-ALL cells. Statistical significance was determined by (d) a two-way repeated measures one-way ANOVA with the Holm-Sidak correction; *P*-values: *<0.05, **<0.01. ns, not significant.

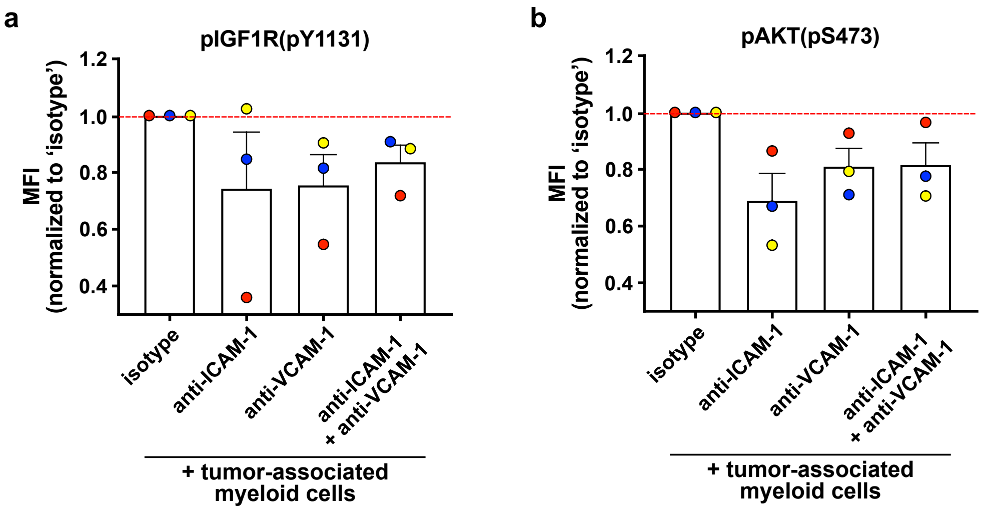

Supplementary Figure 6. Inhibition of ICAM-1- and/or VCAM-1-mediated cell adhesion reduces IGF1R and AKT activation in T-ALL cells.
(a-b) Quantification of (a) pIGF1R and (b) pAKT levels in transplanted LN3 T-ALL cells co-cultured with tumor-associated myeloid cells for 4-5 days in the presence of anti-ICAM-1 and/or anti-VCAM-1 blocking antibodies (10 µg/ml per each) or isotype controls. Results were normalized to the MFI of isotype-treated cultures within each experiment. Bars represent the mean + SEM from n=3 independent experiments, each with a distinct color-coded primary T-ALL. The red line indicates the normalized mean MFI of isotype-treated cultures.

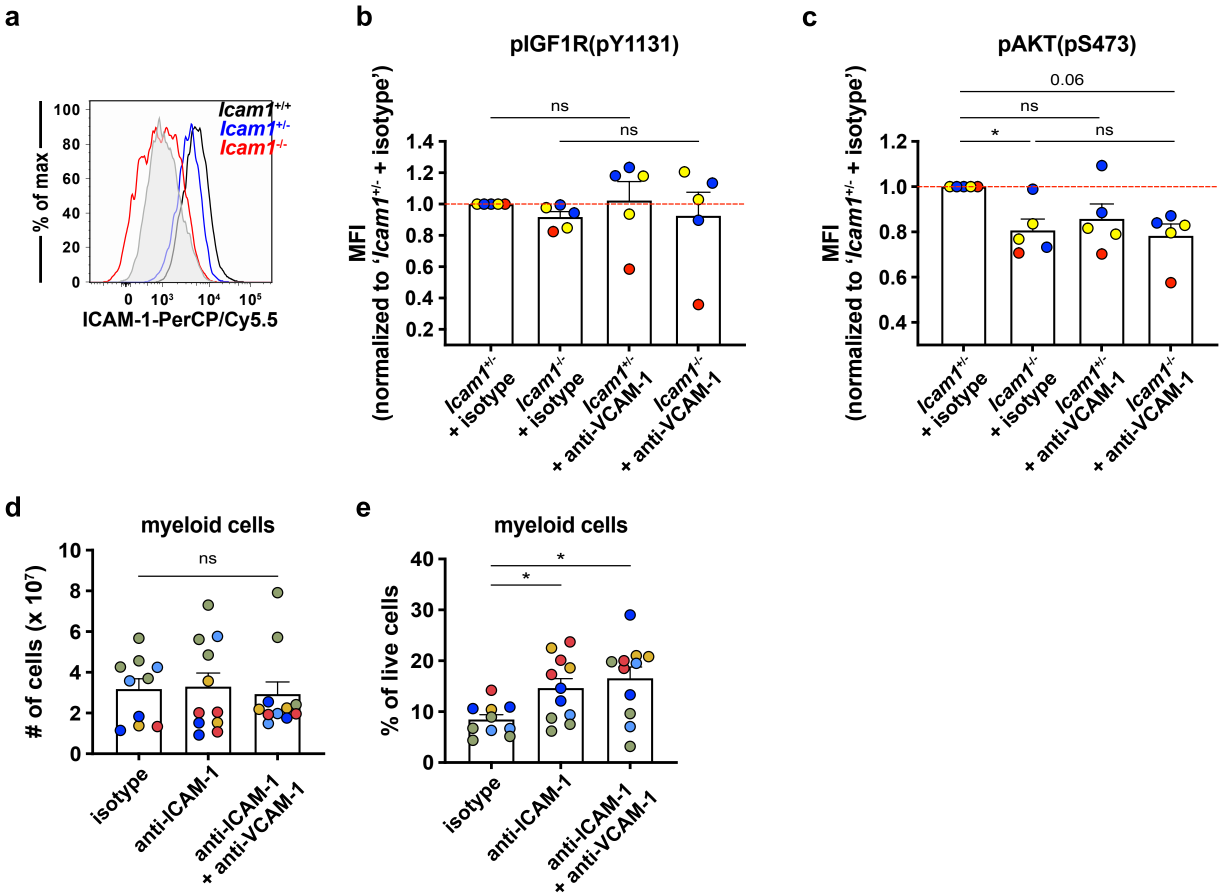

Supplementary Figure 7. Blockade of adhesion molecules in leukemic mice reduces IGF1R and AKT activation but does not reduce myeloid cellularity in the spleen.

(a) Representative flow cytometry histograms of ICAM-1 expression by macrophages ($F4/80^{+}CD64^{+}$) from the spleens of *Icam1*$^{+/+}$ (black), *Icam1*$^{+/-}$ (blue), or *Icam1*$^{-/-}$ (red) mice transplanted with primary LN3 T-ALL cells. Isotype control stains are shaded in gray. (b-c) Quantification of (b) pIGF1R and (c) pAKT levels in T-ALL cells from the spleens of *Icam1*$^{+/-}$ and *Icam1*$^{-/-}$ mice transplanted with primary LN3 T-ALL and treated with anti-VCAM-1 or isotype control antibodies from the same experiments as in Figure 3h. Results were normalized to the MFI of isotype-treated *Icam1*$^{+/-}$ mice within each experiment. Bars represent the mean + SEM from n=3 independent experiments, each with a distinct color-coded primary T-ALL. The red line indicates the normalized mean MFI of isotype-treated *Icam1*$^{+/-}$ mice. (d-e) Quantification of the (d) number and (e) frequency of myeloid cells in the spleens of LN3 T-ALL-transplanted mice following treatment with anti-ICAM-1, anti-ICAM-1 and anti-VCAM-1 (100 μg each per mouse), or relevant isotype control antibodies. Bars depict the mean + SEM of cumulative data from n=5 experiments, each with a distinct color-coded primary T-ALL; symbols represent individual mice. Statistical significance was determined by (b, c, d, e) a two-way repeated measures one-way ANOVA with the Holm-Sidak correction; *P*-values: *<0.05. ns, not significant.

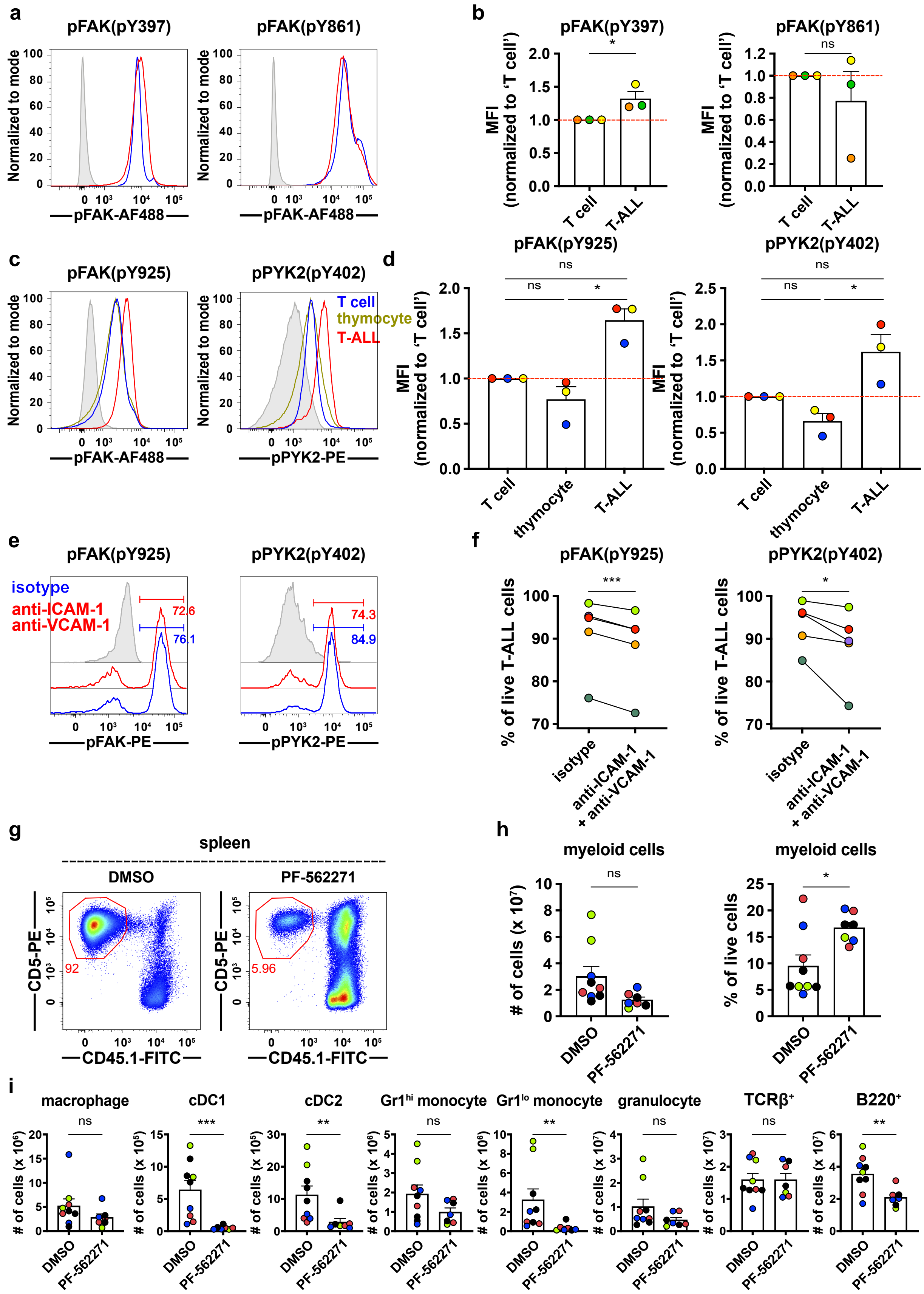

a
pFAK(pY397)
pFAK(pY861)
Normalized to mode
pFAK-AF488
b
MFI (normalized to 'T cell')
T cell
T-ALL
ns
c
pFAK(pY925)
pPYK2(pY402)
T cell
thymocyte
T-ALL
pPYK2-PE
d
e
isotype
anti-ICAM-1
anti-VCAM-1
72.6
76.1
74.3
84.9
pFAK-PE
f
% of live T-ALL cells
anti-ICAM-1 + anti-VCAM-1
g
spleen
DMSO
PF-562271
CD5-PE
CD45.1-FITC
92
5.96
h
myeloid cells
# of cells (x 10⁷)
% of live cells
i
macrophage
cDC1
cDC2
Gr1hi monocyte
Gr1lo monocyte
granulocyte
TCRβ+
B220+
# of cells

Supplementary Figure 8. FAK and PYK2 activation is elevated in T-ALL cells and inhibition of FAK and PYK2 signaling reduces leukemia burden and depletes some myeloid subsets in vivo.

(a-b) (a) Representative flow cytometry plots showing levels of pFAK (pY397 and pY861) in transplanted LN3 T-ALL cells (red) and host T cells (blue) from the same leukemic spleens. Isotype control stains are shaded in gray. (b) Quantification of pFAK levels in T-ALL cells ($CD45.2^+CD5^+$) relative to host T cells ($CD45.1^+CD5^+$), as in (a). Results were normalized to MFIs of T cells within each experiment. Bars represent the mean + SEM from n=3 independent experiments, each with a distinct color-coded primary T-ALL. The red line indicates the normalized mean MFI in T cells. (c-d) (c) Representative flow cytometry plots showing levels of pFAK and pPYK2 in transplanted LN3 T-ALL cells (red), healthy splenic T cells (blue) and healthy thymocytes (green). Isotype control stains are shaded in gray. (d) Quantification of pFAK and pPYK2 levels in T-ALL cells relative to healthy T-cell controls, as in (c). Results were normalized to MFIs of splenic T cells within each experiment. Bars represent the mean + SEM from n=3 independent experiments, each with a distinct color-coded primary T-ALL. The red line indicates the normalized mean MFI in splenic T cells. (e) Representative flow cytometry histograms of pFAK (left) and pPYK2 (right) expression in LN3 T-ALL cells 3-4 days after co-culture with tumor-associated myeloid cells in the presence of blocking antibodies against ICAM-1 and VCAM-1 (red) or isotype controls (blue). Isotype control stains are shaded in gray. (f) Quantification of the frequency of viable T-ALL cells expressing $pFAK^+$ or $pPYK2^+$, as indicated, from n=5 independent experiments as in (e). (g) Representative flow cytometry plots showing a decrease in T-ALL burden in the spleens of LN3 T-ALL-transplanted mice treated as per Figure 5E with PF-562271 (a FAK/PYK2 dual inhibitor; 25 mg per kg body weight) or DMSO. (h) Quantification of the number (left) and frequency (right) of myeloid cells ($CD11b^+$ and/or $CD11c^+$ cells) in the spleens of LN3 leukemic mice following treatment with either PF-562271 or DMSO, in n=4 independent experiments, as in (g). (i) Quantification of the indicated splenic myeloid subsets or $TCR\beta^+$ or $B220^+$ host cells from the same experiments as in (g-h). Data in (g-i) are from the same experiments as in Figure 6b-c. Statistical significance was determined by (b, f) two-way paired Student t tests, (d) a two-way repeated measures one-way ANOVA with the Holm-Sidak correction, and (h, i) two-way unpaired Student t tests; *P*-values: *<0.05, **<0.01, ***<0.001. ns, not significant.

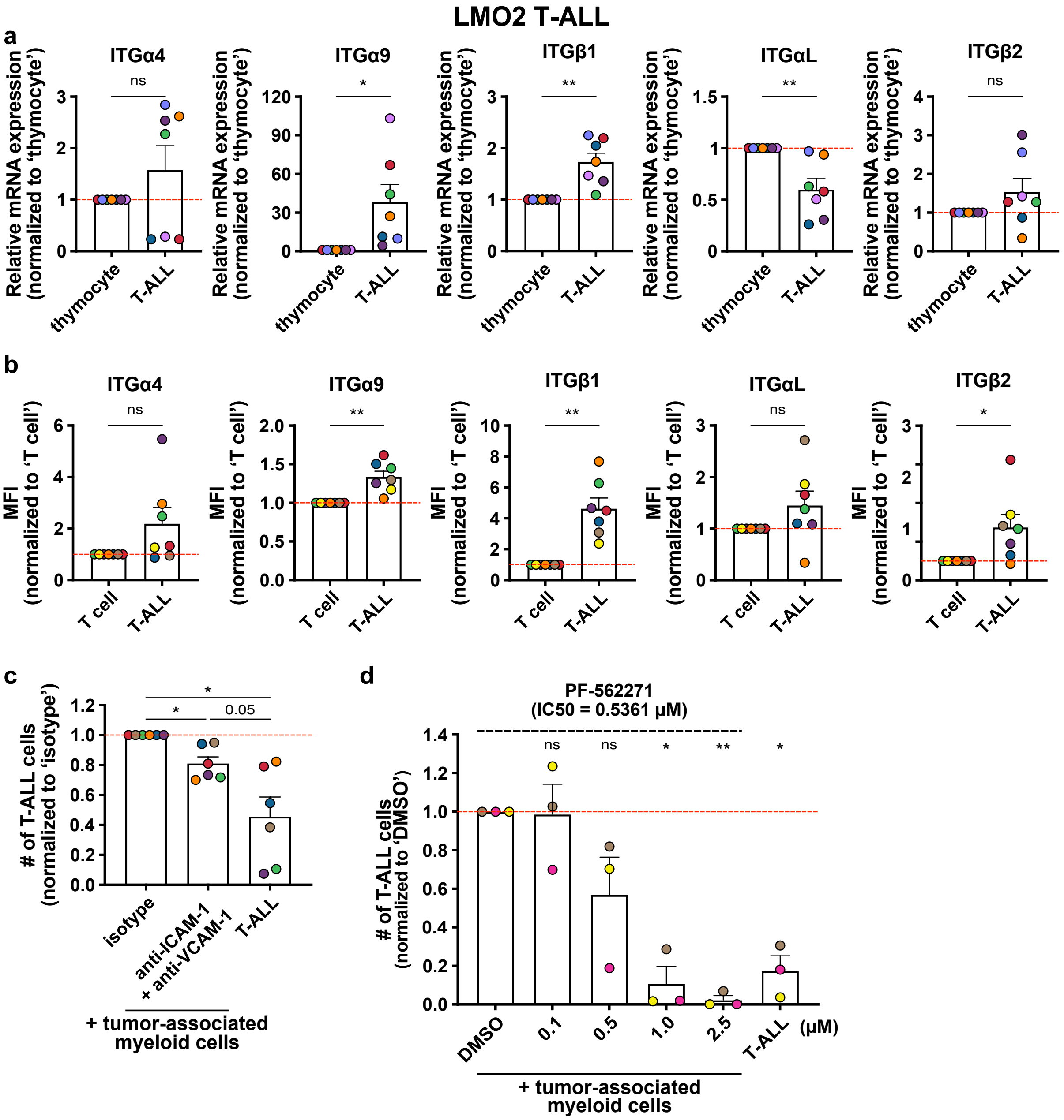
LMO2 T-ALL
a
ITGα4
ITGα9
ITGβ1
ITGαL
ITGβ2
Relative mRNA expression (normalized to 'thymocyte')
thymocyte
T-ALL
ns
*
**
b
MFI (normalized to 'T cell')
T cell
c
# of T-ALL cells (normalized to 'isotype')
isotype
anti-ICAM-1 + anti-VCAM-1
0.05
+ tumor-associated myeloid cells
d
PF-562271 (IC50 = 0.5361 μM)
# of T-ALL cells (normalized to 'DMSO')
DMSO
(μM)

Supplementary Figure 9. Tumor-associated myeloid cells support T-ALL survival in an integrin-dependent manner in the LMO2 mouse of T-ALL.

(a) Relative transcript levels of the indicated integrin subunits were quantified by qRT-PCR in healthy thymocytes and in primary thymic LMO2 T-ALL cells. Results were normalized to the expression level of tumor-free thymocytes within each experiment. Bars represent mean + SEM from n=7 independent experiments, each with a distinct color-coded primary T-ALL. The red line indicates the normalized mean levels of tumor-free thymocytes. (b) Protein expression of the indicated integrin subunits was quantified by flow cytometry on transplanted LMO2 T-ALL cells ($CD45.2^{+}CD5^{+}$) and host T cells ($CD45.1^{+}CD5^{+}$) from leukemic spleens. MFI intensity values were normalized to the MFI of host T cells within each experiment. Data are compiled from n=7 independent experiments, each with a distinct color-coded primary LMO2 T-ALL. (c) Quantification of viable splenic LMO2 T-ALL cells was assessed 6-7 days after culture alone or in the presence of enriched tumor-associated myeloid cells and anti-ICAM-1/anti-VCAM-1 antibodies (10 µg/ml per each) or isotype controls, as indicated. Results were normalized to isotype-treated cultures. Bars show the mean + SEM from n=6 independent experiments, each with a distinct color-coded primary T-ALL; symbols represent the average of 2-3 technical replicates per experiment. The red line indicates the normalized mean viability of isotype-treated T-ALL cells. (d) Quantification of viable LMO2 T-ALL cells co-cultured with enriched tumor-associated myeloid cells for 4 days before addition of the indicated concentrations of a FAK/PYK2 dual inhibitor (PF-562271) or vehicle control (DMSO). Viability was assessed after 2-3 days. Results were normalized to DMSO-treated cultures in each experiment. Bars represent means + SEM from n=3 independent experiments using distinct color-coded primary T-ALLs; symbols represent the average of 2-3 technical replicate wells. The red line indicates the normalized mean T-ALL viability in DMSO-treated cultures. Statistical significance was determined by (a, b) two-way paired Student t tests, and (c, d) a two-way repeated measures one-way ANOVA with the Holm-Sidak correction; *P*-values: *<0.05, **<0.01. ns, not significant.

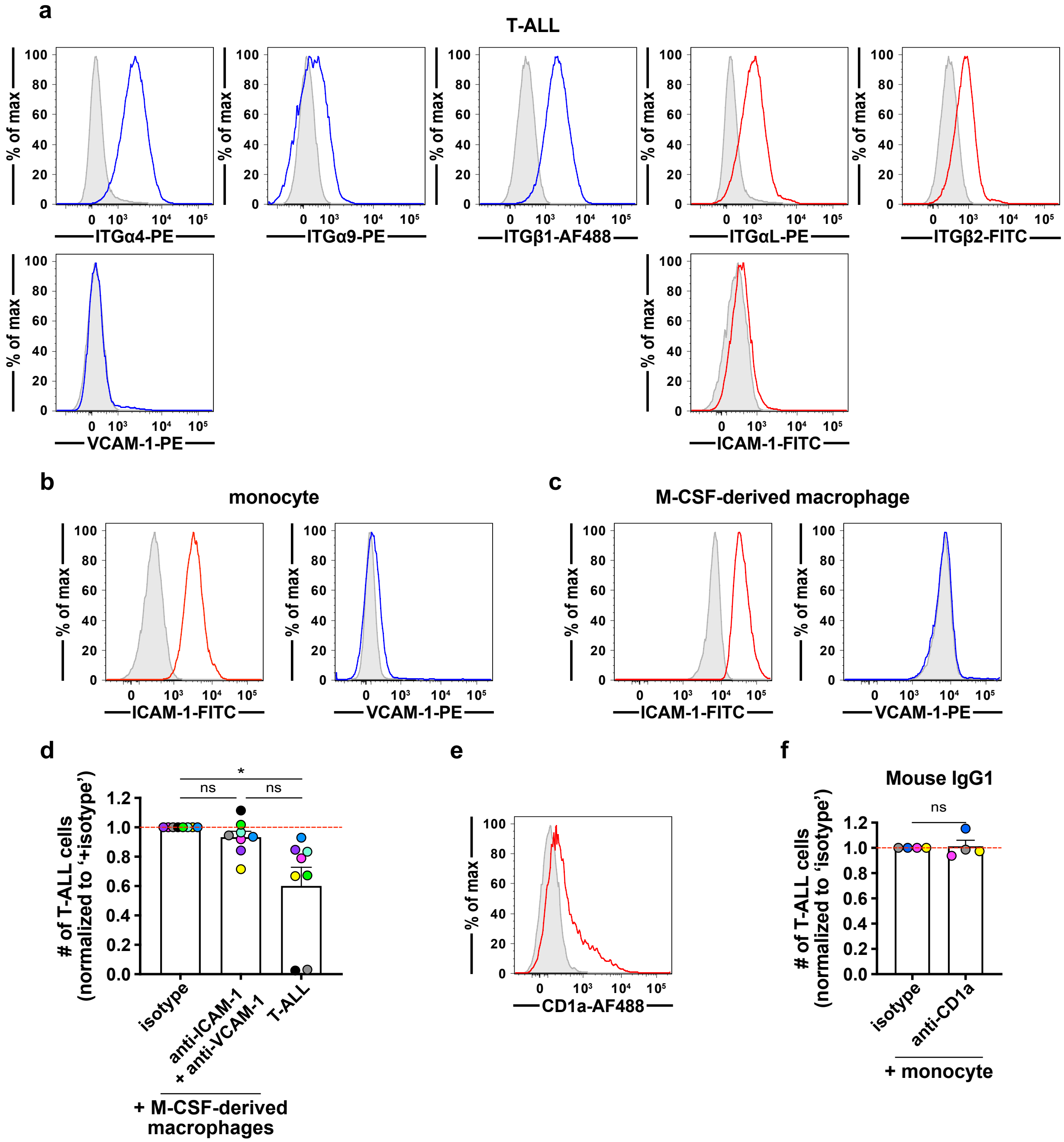
a
T-ALL
% of max
ITGα4-PE
ITGα9-PE
ITGβ1-AF488
ITGαL-PE
ITGβ2-FITC
VCAM-1-PE
ICAM-1-FITC
b
monocyte
ICAM-1-FITC
VCAM-1-PE
c
M-CSF-derived macrophage
ICAM-1-FITC
VCAM-1-PE
d
# of T-ALL cells (normalized to '+isotype')
isotype
anti-ICAM-1 + anti-VCAM-1
T-ALL
+ M-CSF-derived macrophages
ns
*
e
CD1a-AF488
f
Mouse IgG1
# of T-ALL cells (normalized to 'isotype')
isotype
anti-CD1a
+ monocyte

Supplementary Figure 10. Integrins and adhesion molecules are expressed by primary patient T-ALL cells and human myeloid cells, respectively, and diminished T-ALL survival in myeloid co-cultures in the presence of anti-integrin antibodies does not reflect opsonization.
(a) Representative flow cytometry histograms of the indicated integrin components or adhesion molecules present on primary patient T-ALL cells. Integrin components binding to VCAM-1 and ICAM-1 are shown in blue and red, respectively. Isotype control stains are shaded in gray. (b-c) Representative flow cytometry histograms showing ICAM-1 (red) and VCAM-1 (blue) expression by (b) PBMC-derived monocytes and (c) M-CSF-derived macrophages. Isotype control stains are shaded in gray. (d) Quantification of viable primary patient T-ALL cells cultured for 6-7 days alone or with M-CSF-derived macrophages. Co-cultures were carried out in the presence of (a) anti-ICAM-1 (20 μg/ml) and anti-VCAM-1 (10 μg/ml) blocking antibodies or isotype control antibodies as indicated. Results were normalized to isotype-treated cultures in each experiment. Bars represent means + SEM from n=7 independent experiments using 8 distinct, color-coded patient-derived T-ALLs; symbols represent the average of 2 technical replicate wells. The red line indicates the normalized mean T-ALL viability in isotype-treated cultures. (e) Representative flow cytometry plots of CD1a expression by primary patient T-ALL cells. Isotype control stain is shaded in gray. (f) Quantification of viable patient T-ALL cells 6-7 days after co-culture with PBMC-derived monocytes in the presence of anti-CD1a (Mouse IgG1; 30 μg/ml). Results were normalized to isotype-treated cultures in each experiment. Bars represent means + SEM from n=4 independent experiments using distinct, color-coded patient-derived T-ALLs; symbols represent the average of 2-3 technical replicate wells. The red line indicates the normalized mean T-ALL viability in isotype-treated cultures. Statistical significance was determined by (d) a two-way repeated measures one-way ANOVA with the Holm-Sidak correction, (f) two-way paired Student t tests. *P*-values: *<0.05. ns, not significant.
