# Supplementary material for: Integrin signaling is critical for myeloid-mediated support of T-cell acute lymphoblastic leukemia
Source: Nat Commun. 2023 Oct 7;14:6270. doi: 10.1038/s41467-023-41925-z (PMC10560206; doi:10.1038/s41467-023-41925-z)
Supplement: Supplementary file 2 — Reporting Summary [file 41467_2023_41925_MOESM2_ESM.pdf]

nature portfolio

Corresponding author(s): Lauren I. R. Ehrlich

Last updated by author(s): Aug 20, 2023

Reporting Summary

Nature Portfolio wishes to improve the reproducibility of the work that we publish. This form provides structure for consistency and transparency in reporting. For further information on Nature Portfolio policies, see our Editorial Policies and the Editorial Policy Checklist.

Statistics

For all statistical analyses, confirm that the following items are present in the figure legend, table legend, main text, or Methods section.

n/a	Confirmed	
☐	☒	The exact sample size (*n*) for each experimental group/condition, given as a discrete number and unit of measurement
☐	☒	A statement on whether measurements were taken from distinct samples or whether the same sample was measured repeatedly
☐	☒	The statistical test(s) used AND whether they are one- or two-sided<br>*Only common tests should be described solely by name; describe more complex techniques in the Methods section.*
☒	☐	A description of all covariates tested
☐	☒	A description of any assumptions or corrections, such as tests of normality and adjustment for multiple comparisons
☐	☒	A full description of the statistical parameters including central tendency (e.g. means) or other basic estimates (e.g. regression coefficient) AND variation (e.g. standard deviation) or associated estimates of uncertainty (e.g. confidence intervals)
☐	☒	For null hypothesis testing, the test statistic (e.g. *F*, *t*, *r*) with confidence intervals, effect sizes, degrees of freedom and *P* value noted<br>*Give P values as exact values whenever suitable.*
☒	☐	For Bayesian analysis, information on the choice of priors and Markov chain Monte Carlo settings
☒	☐	For hierarchical and complex designs, identification of the appropriate level for tests and full reporting of outcomes
☐	☒	Estimates of effect sizes (e.g. Cohen's *d*, Pearson's *r*), indicating how they were calculated

Our web collection on statistics for biologists contains articles on many of the points above.

Software and code

Policy information about availability of computer code

Data collection	flow cytometry: BD FACSDiva (multiple versions due to software updates)
Data analysis	flow cytometry: FlowJo (Tree Star, Inc.; v9 and v10, multiple versions due to software updates)<br>RNA-seq: Kallisto (v0.46.1), HISAT2 (v2.0.4), R packages (multiple versions due to software updates for each) including DESeq2, msigdbr, fgsea, and biomaRt, and Enrichr (https://maayanlab.cloud/Enrichr/)<br>statistical testing: Prism (GraphPad Software; v9, multiple versions due to software updates)

For manuscripts utilizing custom algorithms or software that are central to the research but not yet described in published literature, software must be made available to editors and reviewers. We strongly encourage code deposition in a community repository (e.g. GitHub). See the Nature Portfolio guidelines for submitting code & software for further information.

Data

Policy information about availability of data

All manuscripts must include a data availability statement. This statement should provide the following information, where applicable:

- Accession codes, unique identifiers, or web links for publicly available datasets
- A description of any restrictions on data availability
- For clinical datasets or third party data, please ensure that the statement adheres to our policy

The data generated in this study are available within the article and its supplementary data files, and source data are provided with this paper. The mouse RNA-seq

data analyzed in this study were obtained from Gene Expression Omnibus (GEO) at GSE150096 [https://www.ncbi.nlm.nih.gov/geo/query/acc.cgi?acc=GSE150096]. The patient RNA-seq data analyzed in this study were obtained from the TARGET website at https://ocg.cancer.gov/programs/target. The transcriptomics data of FAK-WT and FAK-null murine squamous cell carcinoma cells were obtained from GEO at GSE147670 [https://www.ncbi.nlm.nih.gov/geo/query/acc.cgi?acc=GSE147670].

Research involving human participants, their data, or biological material

Policy information about studies with human participants or human data. See also policy information about sex, gender (identity/presentation), and sexual orientation and race, ethnicity and racism.

Reporting on sex and gender	The researchers are not reporting on sex and gender. However, both sexes were recruited.
Reporting on race, ethnicity, or other socially relevant groupings	The researchers are not reporting on race, ethnicity, or other socially relevant groupings. However, for ethnicity, both hispanic and non-hispanic participants were recruited.; for race, both caucasians and Black Americans were recruited.
Population characteristics	For experiments in which human T-ALL cells were grown in cell culture, de-identified primary pediatric T-ALL samples were used. Partcipants who provided biospecimens used in experiments ranged in age from 2-13 years, were black or caucasian, and hispanic or non-hispanic. All but one were male.
Recruitment	De-identified patient primary pediatric T-ALL samples were obtained from Texas Children's Hospital (Houston, TX). Informed consent from the parent and assent from children over the age of 8 years were obtained.
Ethics oversight	Sample procurement and analysis were approved by the institutional review board committees at The University of Texas at Austin and Texas Children's Hospital/Baylor College of Medicine.

Note that full information on the approval of the study protocol must also be provided in the manuscript.

Field-specific reporting

Please select the one below that is the best fit for your research. If you are not sure, read the appropriate sections before making your selection.

[x] Life sciences   [ ] Behavioural & social sciences   [ ] Ecological, evolutionary & environmental sciences

For a reference copy of the document with all sections, see nature.com/documents/nr-reporting-summary-flat.pdf

Life sciences study design

All studies must disclose on these points even when the disclosure is negative.

Sample size	A minimum of 3 experiments were conducted per each in vitro or in vivo experiment. The number of experiments was chosen based on analysis of comparable data in our previous reports, including Lyu et al., Blood and Triplett et al., PNAS, variance in preliminary data, and also based on previous literature.
Data exclusions	No data were excluded from the analyses.
Replication	At least three independent biological replications, which were successful, were carried out for each experiment.
Randomization	Littermate mice were used for engraftment of primary T-ALL cells, controlling for variations in mouse background and age. When possible, mice were sex-matched, depending on the size of the litter. Upon established T-ALL burden, mice were randomly divided into experimental and control groups.
Blinding	The investigators were blinded to group allocation during data collection and data analysis.

Reporting for specific materials, systems and methods

We require information from authors about some types of materials, experimental systems and methods used in many studies. Here, indicate whether each material, system or method listed is relevant to your study. If you are not sure if a list item applies to your research, read the appropriate section before selecting a response.

Materials & experimental systems

n/a	Involved in the study
☐	☒ Antibodies
☒	☐ Eukaryotic cell lines
☒	☐ Palaeontology and archaeology
☐	☒ Animals and other organisms
☒	☐ Clinical data
☒	☐ Dual use research of concern
☒	☐ Plants

Methods

n/a	Involved in the study
☒	☐ ChIP-seq
☐	☒ Flow cytometry
☒	☐ MRI-based neuroimaging

Antibodies

Antibodies used

For mouse lymphoid staining, anti-mouse CD4-PerCP/Cy5.5 (RM4-5), CD5-PE (53-7.3), CD8-Pacific Blue (53-6.7), CD45.1-FITC (A20), CD45.2-APC/Cy7 (104), B220-Alexa Fluor 700 (RA3-6B2), and TCRβ-PE/Cy7 (H57-597) antibodies were used. For mouse myeloid staining, CD64-PE (X54-5/7.1), CD11c-Pacific Blue (N418), I-A/I-E-APC/Cy7 (M5/114.15.2), CD172a-PE/Cy7 (SIRPα; P84), biotinylated XCR1 (ZET), CD11b-Alexa Fluor 700 (M1/70), CD115-Alexa Fluor 488 (AFS98), Gr1-Brilliant Violet 510 (RB6-8C5), F4/80-Alexa Fluor 647 (T45-2342; BD Biosciences), and Qdot 605 streptavidin conjugate (Invitrogen) antibodies were used. For mouse integrin staining, anti-mouse CD11a-FITC (ITGαL; 2D7), CD18-PE (ITGβ2; M18/2), CD29-PE (ITGβ1; HMβ1-1), CD49d-Alexa Fluor 488 (ITGα4; R1-2), Integrin α9 (N-19; Santa Cruz Biotechnology), and donkey anti-goat IgG-Alexa Fluor 488 (Jackson ImmunoResearch) antibodies were used. For mouse adhesion molecule staining, anti-mouse CD54-PerCP/Cy5.5 (ICAM-1; YN1/1.7.4) and CD106-PerCP/Cy5.5 (VCAM-1; 429(MVCAM.A)) antibodies were used. The relevant isotype antibodies (RTK2758, RTK4530, HTK888, and Poly24030) were used as controls.

For human myeloid staining, anti-human CD11b-Alexa Fluor 700 (M1/70) and CD14-PerCP/Cyanine5.5 (M5E2) were used. For human integrin staining, anti-human CD11a-PE (HI111), CD18-FITC (TS1/18), CD29-Alexa Fluor 488 (TS2/16), CD49d-PE (9F10), and Integrin α9β1-PE (Y9A2) antibodies were used. For human adhesion molecule staining, anti-human CD54-FITC (HA58), CD106-APC or -PE (STA), CD106 (AF809; R&D Systems), and donkey anti-sheep IgG-Alexa Fluor 488 (Invitrogen) antibodies were used. The relevant isotype antibodies (MOPC-21 and 31243 (Invitrogen) were used as controls.

For intracellular immunostaining of proteins, single-cell suspensions were labeled with LIVE/DEAD Fixable Dead Cell Stain Kit (Green or Aqua; Invitrogen) and then treated with FIX & PERM Fixation & Cell Permeabilization Kit (Invitrogen) according to the manufacturer's methanol modification procedure. Cells were then stained with fluorescently labeled antibodies against anti-mouse CD45.1-PerCP/Cy5.5 (A20), CD45.2-APC/Cy7 (104), CD5-PE/Cy7 (53-7.3), pIGF1R-Alexa Fluor 647 (K74-218; pY1131; BD Biosciences), pAKT-PE or -Brilliant Violet 421 (J24-618; pS473; BD Biosciences), pPYK2-PE (L68-1256.272; pY402; BD Biosciences), pFAK (#3284; pY925; Cell Signaling Technology), donkey anti-rabbit IgG-PE (Poly4064), ILK (P83A9), and goat anti-mouse IgG-Alexa Fluor 488 (Invitrogen) antibodies. The relevant isotype antibodies (MOPC-21, MPC-11, and Poly29108) were used as negative controls.

Validation

All antibodies used are commercially available, and were validated by the manufacturer. Upon receipt, antibodies were tested in the laboratory using known positive and negative controls.

Animals and other research organisms

Policy information about studies involving animals; ARRIVE guidelines recommended for reporting animal research, and Sex and Gender in Research

Laboratory animals

For primary T-ALL, LN3 and LMO2 mice were used. For engraftment of T-ALL cells, B6.129S4-Icam1tm1Jcgr/J (Icam1-/-), C57BL/6J, and B6.SJL-Ptprca Pepcb/BoyJ (CD45.1) mouse strains were used. Recipient mice were 6-8 weeks of age. Mice were housed in a 12-hour dark/light cycle, in ventilated cages, under regularly monitored temperature (68-76°F) by veterinary staff.

Wild animals

No wild animals were used in this study.

Reporting on sex

This study does make any conclusions reporting on sex. Both sexes of recipient mice were used as we have not observed sex-based differences in T-ALL engraftment, progression, or myeloid dependence.

Field-collected samples

No field-collected samples were used in this study..

Ethics oversight

All experimental procedures were approved by the Institutional Animal Care and Use Committee at The University of Texas at Austin.

Note that full information on the approval of the study protocol must also be provided in the manuscript.

Flow Cytometry

Plots

Confirm that:

- [x] The axis labels state the marker and fluorochrome used (e.g. CD4-FITC).
- [x] The axis scales are clearly visible. Include numbers along axes only for bottom left plot of group (a 'group' is an analysis of identical markers).
- [x] All plots are contour plots with outliers or pseudocolor plots.
- [x] A numerical value for number of cells or percentage (with statistics) is provided.

Methodology

Sample preparation	Organs were harvested and processed as follows: Spleens and thymi were mechanically dissociated with FACS wash buffer (FWB: phosphate-buffered saline (PBS) supplemented with 2% (v/v) BCS (Bovine Calf Serum; GemCell) and 0.5 mM EDTA). Bone marrow cells were obtained by flushing femurs with 2 ml of FWB. Inguinal lymph nodes were enzymatically digested with a cocktail of 0.6 mg/ml (w/v) Liberase and 20 U/ml DNase I (both from Roche). LNs were sequentially digested 3 times with 2 ml of cocktail for 12 minutes per digest. Livers were mechanically dissociated, spun at 50 g for 3 minutes, and the supernatant was collected and analyzed. All single-cell suspensions were filtered using 40 µm filters (Fisher Scientific) and subjected to red blood cell lysis using RBC Lysis Buffer (BioLegend). Cells were immunostained by incubating at 4˚C for 30 minutes with fluorescently labeled antibodies below (all antibodies were purchased from BioLegend, unless otherwise indicated). After staining, cells were washed 1-2 times in FWB and resuspended in FWB containing 1 µg/ml propidium iodide (PI; Enzo Life Sciences) to assess viability.
Instrument	BD LSR II flow cytometer
Software	BD FACSDiva for collection, Tree Star FlowJo for analysis
Cell population abundance	Tumor-associated myeloid cells were positively enriched to >70% using biotinylated antibodies against CD11c and streptavidin beads (BioLegend). T-ALL cells were negatively enriched to >99% following depletion of cells expressing F4/80, CD11b, I-A/I-E, and/or CD11c.
Gating strategy	Cells were initially gated based on size FSC/SSC, followed by size FSC to exclude cell doublets. Live cells were then gated based on viability dye before analysis for cellular markers as shown in supplemental materials.

- [x] Tick this box to confirm that a figure exemplifying the gating strategy is provided in the Supplementary Information.
